# Large-scale literature mining to assess the relation between anti-cancer drugs and cancer types

**DOI:** 10.1186/s12967-021-02941-z

**Published:** 2021-06-26

**Authors:** Chris Bauer, Ralf Herwig, Matthias Lienhard, Paul Prasse, Tobias Scheffer, Johannes Schuchhardt

**Affiliations:** 1 MicroDiscovery GmbH, Marienburger Straße 1, 10405 Berlin, Germany; 2 Department of Computational Molecular Biology, Max Planck Institute for Molecular Genetics, Ihnestraße 63, 14195 Berlin, Germany; 3 Department of Informatics, University of Potsdam, August-Bebel-Str. 89, 14482 Potsdam, Germany

**Keywords:** Literature mining, Anti-cancer drugs, Tumor types, Word embeddings, Database

## Abstract

**Background:**

There is a huge body of scientific literature describing the relation between tumor types and anti-cancer drugs. The vast amount of scientific literature makes it impossible for researchers and physicians to extract all relevant information manually.

**Methods:**

In order to cope with the large amount of literature we applied an automated text mining approach to assess the relations between 30 most frequent cancer types and 270 anti-cancer drugs. We applied two different approaches, a classical text mining based on named entity recognition and an AI-based approach employing word embeddings. The consistency of literature mining results was validated with 3 independent methods: first, using data from FDA approvals, second, using experimentally measured IC-50 cell line data and third, using clinical patient survival data.

**Results:**

We demonstrated that the automated text mining was able to successfully assess the relation between cancer types and anti-cancer drugs. All validation methods showed a good correspondence between the results from literature mining and independent confirmatory approaches. The relation between most frequent cancer types and drugs employed for their treatment were visualized in a large heatmap. All results are accessible in an interactive web-based knowledge base using the following link: https://knowledgebase.microdiscovery.de/heatmap.

**Conclusions:**

Our approach is able to assess the relations between compounds and cancer types in an automated manner. Both, cancer types and compounds could be grouped into different clusters. Researchers can use the interactive knowledge base to inspect the presented results and follow their own research questions, for example the identification of novel indication areas for known drugs.

**Supplementary Information:**

The online version contains supplementary material available at 10.1186/s12967-021-02941-z.

## Background

Cancer is one of the leading causes of mortality with an estimated number of 18.1M cases and 9.6M deaths in 2018 [1]. For the chemotherapeutic or targeted treatment of cancer, there is a large number of anti-cancer drugs available. 156 anti-cancer drugs were approved by the FDA (from 1989 to 2017) [2] but there are many more potential anti-cancer drugs. The NIH website^1^ lists currently more than 600 drugs (generic names and brand names) approved for anti-cancer theraphy. There is a huge number of scientific publications describing the relation between tumor types and anti-cancer drugs (e.g. the effectiveness of a drug for a tumor type). The vast amount of literature makes it impossible for a human to extract all relevant information, even for a specific topic (e.g. search term ‘breast cancer’ results in 258K publications-on August 14, 2019). A second problem arises from inequality of attention a publication receives. While cited articles are receiving more attention and in turn more citations, approximately 15% will most likely never be cited [3].

Most of the scientific knowledge is published as unstructured text. Although these texts are human readable they are not per se machine-interpretable. There are two different kinds of widely used approaches to transform text into machine-interpretable data.

First: classical text mining tools aim to recognize certain types of entities (e.g. genes/proteins [4, 5] or compounds [6, 7]). In addition to specific entities, some tools are also trying to extract relations between those entities e.g. protein–protein interactions [8, 9] or relations between genes and miRNAs [10]. These tools are typically using prior knowledge about entities such as synonyms, trivial names, or ontologies as well as knowledge about semantics (e.g. keywords that imply a certain relation between entities [8]). Other approaches are extracting less clearly defined events such as CHAT, a text mining tool to visualize cancer hallmarks [11]. There are also approaches to integrate several text mining tools in a standardized way, such as iTextMine which offers a web interface to search for relations between genes, miRNAs, diseases, or drugs [12].

Second: Word embeddings are designed to transform the text into machine interpretable numeric vectors. They allow to identify similar words by comparing the word vectors. The two most common implementations are Global Vectors for Word Representation (Glove) [13] and Word2Vec [14]. To learn the word embeddings a large amount of text is required. This large amount of text makes it difficult to analyze rare words. For better learning rare words, approaches with a sub-word embedding model have been proposed [15]. Word embeddings have been applied in material science to demonstrate that an unsupervised method can recommend materials for functional applications several years before their discovery [16]. There are also biomedical application, e.g. for combining sub-word information and biomedical controlled vocabulary (MeSH) [17]. However, there is no consistent global ranking of word embeddings for all downstream biomedical natural language processing applications [18]. Also a comparison between the two approaches (classical text mining vs. word embeddings) is difficult as they are quite different in nature.

In this manuscript, we aim to automatically assess the relations between the 30 most frequent cancer types and 270 anti-cancer drugs using biomedical publications. We apply two different text mining strategies, first a supervised approach with classical text mining strategies and second an unsupervised approach based on the calculation of word embeddings. To assess the relation between cancer types and anti-cancer drugs we downloaded and analyzed almost 4 million publications (abstracts from PubMed). We demonstrate that an automated text mining is able to assess the relation between cancer types and anti-cancer drugs. The relevance of the extracted relations was confirmed using three independent methods: First, by the comparison with FDA approval information, second, by using IC-50 values from a huge experimental dataset of compounds and cancer cell lines [19] and third, by the comparison with clinical survival data. To our knowledge this is the first large scale application of literature mining to characterize the relation between compounds and cancer types.

In addition, we make the results of the analyses accessible in an interactive web-based knowledge base. The knowledge base enables researchers and clinicians to investigate relations between a certain combination of cancer type and compound very efficiently. We are not aware of other available tools with a similar scope.

## Methods

### Selection of publications

For retrieving cancer type related publications we selected the 30 most frequent cancer types (world wide for both sexes) according to the World Cancer Research Fund International^2^. For each cancer type we manually defined a list of synonyms (e.g. for stomach cancer: ‘stomach cancer’, ‘cancer of the stomach’ or ’gastric cancer’). The full list is provided as Additional file 1. These synonyms were then used to build a PubMed query by concatenating the synonyms with a logical ‘OR’. The abstracts of the corresponding publication where downloaded as XML-files using the R package easyPubMed (version 2.11). For the 30 tumor types we downloaded abstracts from a total of $\sim$ 2.4M publications.

For retrieving compound related publications we selected a set of 266 compounds from cancerrxgene archive (Genomics of Drug Sensitivity in Cancer) [19] (RRID:SCR_011956). Furthermore we included the 24 compounds used in the Cancer Cell Line Encyclopedia (CCEL) [20] (RRID:SCR_013836). This resulted in a total number of 270 compounds. For each compound we retrieved all known synonyms by using the PubChem web service [21] (RRID:SCR_004284). We tested each synonym for any results from the PubMed database (RRID:SCR_004846) and removed synonyms if they do not show any result. All synonyms for which we found any publications were then used to build a query by concatenating the synonyms with a logical ’OR’. The abstracts of the corresponding publications where downloaded as XML-files using R package easyPubMed. For the 270 compounds we downloaded a total of $\sim$ 1.3M publication abstracts.

Furthermore we downloaded all abstracts as XML files from 1996 to 2019 using the PubMed web interface. This reference set is used to calculate the significance of a set of publications based on the Fisher test (detailed examples of the Fisher test are given later for the extraction of genes and the relation between compounds and tumor types). The reference set includes more than 30M publications.

Please note: The keyword search was performed using a PubMed query (with standard settings). As an effect the keywords must not explicitly occur in the title or abstract since PubMed used Automatic Term Mapping (ATM). E.g. the search for ’cervical cancer’ results in the following detailed query: “uterine cervical neoplasms”[MeSH Terms] OR (“uterine”[All Fields] AND “cervical”[All Fields] AND “neoplasms”[All Fields]) OR “uterine cervical neoplasms”[All Fields] OR (“cervical”[All Fields] AND “cancer”[All Fields]) OR “cervical cancer”[All Fields].

### Extraction of genes/proteins from literature

To extract genes/proteins from literature is to apply an algorithm for named entity recognition (NER). For gene/protein NER and normalization we used the GNAT library [4] (version 1.22) with all available filters (see also [5] for a comparions of GNAT and GNormPlus). In literature, there are omni-present genes/proteins occuring in many publications without a specific relation (e.g. Albumin). Since we are not interested in omni-present proteins, we perform an over-representation analysis in order to assess if a gene is specific for a given context (e.g. if a gene is significantly over-represented in a set of publications for a compound or cancer type). For assessing the significance of an association we calculate a Fisher test p-value as well as an odds ratio. With this procedure we extract a ranked list of specific genes/proteins associated with a set of publications.

The calculation of the Fisher test p-value is demonstrated on the example of breast cancer and the two genes/proteins: ’Albumin’ (ALB) and ’Breast cancer type 1 susceptibility protein’ (BRCA1). For breast cancer we extracted $\sim$ 387K publications, $\sim$ 95K of which containing any genes/proteins. Albumin was found in 817 publications, BRCA1 was found in 5912 publications. As reference set we used $\sim$ 30M publications, 2.8M of which containing any genes/proteins. Albumin was found in 55K publications, BRCA1 was found in 9357 publications. This corresponds to a p-value of 1 for the Albumin (not specific for breast cancer) and $<10^{-300}$ (highly specific for breast cancer) for BRCA1 (see contingency tables below).

**Table:** 

	Breast cancer	Reference
Albumin	817	$\sim$54K
Other Prot	$\sim$94K	$\sim$2.7M
	P-value: 1

**Table:** 

	Breast cancer	Reference
BRCA1	5912	3445
Other Prot	$\sim$90K	$\sim$2.7M
	P-value: $<10^{-300}$	

### Assessing the relation between compounds and tumor types

To calculate the significance of the association between a compound and a cancer type we use the Fisher test and perform an over-representation analysis. E.g. for breast cancer or doxorubicin we found 378K publications, 10K of which were found for both. The significance is calculated by comparing to all $\sim$ 30M publications and is highly significant $<10^{-300}$.

The significance for the overlap of genes is calculated in the same way. For breast cancer or doxorubicin we found 759 genes in all publications, 95 genes were found for both. The significance is calculated by comparing to all $\sim$ 15K genes found in all $\sim$ 30M publications and is highly significant $\sim 10^{-82}$.

**Table:** 

Overlap of publications		
	Breast cancer	Reference
Doxorubicin	$\sim$10K	$\sim$68K
Reference	$\sim$300K	$\sim$29M
	P-value: $<10^{-300}$	

**Table:** 

Overlap of genes		
	Breast cancer	Reference
Doxorubicin	95	579
Reference	86	$\sim$15K
	P-value: $\sim 10^{-82}$	

### Identification of co-occuring compounds

In order to identify co-occuring compounds for a tumor type, we are using all publications for the corresponding tumor type. For all pairs of compounds we are than using the Fisher test to assess if the co-occurrence of the compounds is statistically significant. The calculation of the statistics is analogously to the extraction of genes and the relation between compounds and tumor types.

The list of co-occuring compounds for a tumor type is than used to build a co-occuring network graph. To this end we are using all pairs of compounds with a $-\text{log}_{10}$ p-value $> 50$ and generate the co-occurrence graph.

### Word embeddings

For the calculation of word embeddings we used the publications downloaded for all tumor types and all compounds. The most common way to calculate numerical vectors for text representation is to use neural networks as implemented in Word2Vec [14]. For the calculation of word embeddings we used the Deeplearning4J (DL4J) platform (version 1.0.0-beta4) [22]. We used the following set of parameters: minimal word frequency = 1 (consider all words); layer size = 200 (length of the word vector); window size = 500 (approximately the number of words per abstract). To calculate the similarity between compounds and cancers we use the cosine distance.

#### Text preprocessing

Typically word embeddings are working on single words. They are used to identify similar words or synonyms. We want to apply word embeddings to assess similarity between compounds and cancer types (including the known synonyms for both). To this end we replaced all relevant synonyms with a specific compound or cancer ID during the extraction of sentences. E.g.: the phrase *’liver cancer were treated with the same procedure employing 5-FU, mitomycin C, adriamycin’* is transformed to *’cancerlivercancer were treated with the same procedure employing 5-fluorouracil mitomycin-c doxorubicin’*. Note: We used ’cancerlivercancer’ as an internal ID for all synonyms of liver cancer for the calculation of word embeddings.

### Validation

#### FDA approval information

In order to get information about FDA (U.S. Food and Drug Administration, RRID:SCR_012945) approval of a drug for a certain cancer type we used the list published by Sun et al. [23]. The drug annotations from this list where mapped to the drug identifiers used in our analyses based on the information from PubChem web service [21] (RRID:SCR_004284).

#### IC-50 values

IC-50 values for a compound and a tumor type were extracted from the cancerrxgene dataset [19] (Genomics of Drug Sensitivity in Cancer, RRID:SCR_011956). To this end, cell lines where mapped manually to tumor types using the GDSC labels. The final IC-50 value is calculated as the 10% quantile of all corresponding IC-50 values. The 10% quantile is chosen in order to select a low IC-50 value of all cell lines representing the same tumor type (instead of using a minimal value which could lead to a certain instability of the results).

#### Survival data

Patient survival data for different tumor types were downloaded from Broad Institute (RRID:SCR_007073) http://gdac.broadinstitute.org/. We downloaded merged clinical datasets, containing information about survival times and medication for a larger number of patients for each tumor type. The drugs annotations from the clinical datasets was mapped to the drug identifiers used in our analyses via PubChem web service [21] (RRID:SCR_004284).

### Statistics

All statistics were calculated in R (version 3.5.1) (R Project for Statistical Computing, RRID:SCR_001905). Significance of overlaps for sets of publications or sets of genes was calculated with Fisher’s exact test (see above for an example). P-values from Fisher’s test were transformed to $-\text{log}_{10}$ p-values. Kaplan-Meier curves were calculated with the R packages: ’survival’ (version 2.42-6) and ’rms’ (version 5.1-2). Survival curves were compared with Cox proportional hazard regression. P-values of Cox regression are calculated using a log-likelihood test.

## Results

### Relation between tumor types and compounds

#### Common publications

**Fig. 1:**
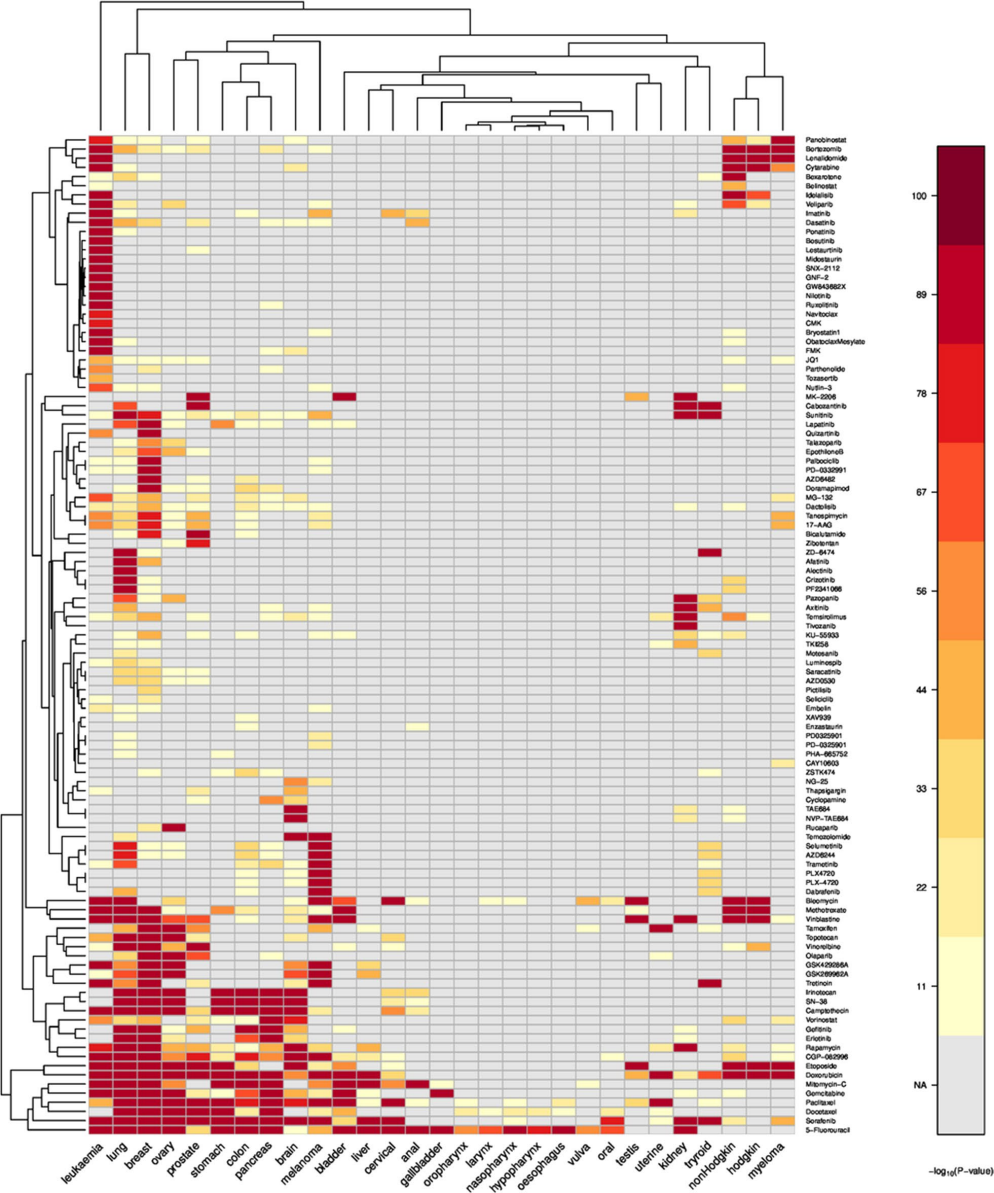
Heatmap overlap of publications. Heatmap visualizing the significance of the overlap between compounds (y-axis) and cancer types (x-axis). The overlap is assessed based on the number of common publications in comparison to the total number of publications ($-\text{log}_{10}$ p-value of Fisher’s test truncated at 100). Compounds showing no significant association to any of the tumor types are not shown

We investigate the relation between 270 compounds and 30 cancer types by assessing the significance of the overlap of common publications compared to the reference. For each combination of compound and cancer-type the significance is calculated with Fisher’s exact test as described in the Methods section. Based on this data we calculate a comprehensive heatmap visualizing the relation between compounds, between cancer types as well as the mutual correspondence as reflected by the literature (see Fig. 1).

This map facilitates to assess the structure in the space of cancer types and compounds. In general, the anti-cancer drugs can be divided in two different groups: On one side there are compounds which are very significantly related to many different (but not all) tumor types such as doxorubicin or 5-fluorouracil. On the other side some compounds are very specifically related to a single cancer type. E.g. nilotinib is exclusively related to leukemia or rucaparib is almost exclusively related to ovary cancer. These two groups typically reflect the different target pathways of the compounds. 13 compounds show very high association ($-\text{log}_{10}$p-value $\ge$ 100) to more than 5 tumor types. These compounds mainly target very general pathways such as ’DNA replication’ (8 compounds) or ’Mitosis’ (3 compounds). On the other side 47 compounds are highly associated to one or two tumor types. These compounds target much more specific (signaling) pathways such as ’RTK signaling’ (9 compounds), ’ERK MAPK signaling’ (7 compounds) or PI3K/MTOR signaling (4 compounds).

A similar grouping also applies for the tumor types. Cancer of pancreas, colon and stomach seem to be similar with respect to the compounds. Although lung and breast cancer have a large list of common compounds, they also show some distinct associations to drugs (e.g. alectinib for lung cancer-approved for treatment of non small cell lung cancer by the FDA on 2017-11-06 or palbociclib in breast cancer [24]). Especially leukemia has a large number of specific compounds.

#### Common molecular descriptor (genes/proteins)

As a variation of the previous approach, we are now adding further knowledge about molecular background such as involved genes/proteins. We perform a similar analysis but focusing on the genes and proteins mentioned in the corresponding publications. For each compound and for each cancer type, we calculate a list of specific genes/proteins (see Methods section). For targeted compounds the top hit is typically the target itself, e.g. for erlotinib (tyrosine kinase inhibitor, acting on the epidermal growth factor receptor) we found a total of 2570 publications containing any gene names, 2164 (84%) of which containing the gene/protein EGFR. As a second example, for ruxolitinib (janus kinase inhibitor) we found a total of 499 publications containing any gene names, 345 (69%) of which containing the gene/protein JAK1 and 191 (38$) containing the gene/protein JAK2. The association between compound and cancer-type is assessed by calculating the significance of the overlap of specific genes (again using Fisher’s test). The association is significant if the overlap of genes extracted from compound related publications and genes extracted from the cancer-type related publications is high compared to the reference of all publications. (see Additional file 2: Figure S1 for the corresponding heatmap).

The number of significant associations is much smaller compared to the previous analysis. The main reason is that less than 20% of the publications contain genes or proteins. However, the $-\text{log}_{10}$ p-values of both approaches are nicely correlated with a Pearson correlation coefficient of $r = 0.64$. Especially the clustering of cancer types is very similar with an adjusted Rand index of $\sim 0.85$. The adjusted Rand index of the compounds is $\sim 0.35$. Again the compounds doxorubicin and 5-fluorouracil are generic and related to many tumor types while nilotinib is only related to leukemia.

#### Word embeddings

In contrast to the two previous approaches word embeddings represent an unsupervised approach. Word vectors are trained using all publications of any cancer and any compound (2.4M + 1.3M) together. Distance matrix of compounds and cancer types is calculated using the cosine similarity of the corresponding word vectors. A high cosine similarity reflects a similar orientation of the corresponding word vectors and does not necessarily mean a direct relation.

The results from unsupervised word embeddings are slightly different from the previous results. The Pearson correlation coefficient between cosine similarity and the $-\text{log}_{10}$ p-values from the overlap of publications is only $r = 0.4$ (see Additional file 3: Figure S2 for the corresponding heatmap). The clustering of cancer types is quite different with an adjusted Rand index of $\sim 0.24$. Also the clustering of compounds is different with an adjusted Rand index of $\sim 0.16$.

### Validation

The primary goal of this validation is to demonstrate that relations between tumor types and compounds extracted by the automatic literature mining are confirmed by different independent sources. In addition we will use the three validation strategies to compare the different literature mining approaches.

#### Comparison with drug approval information

As a first independent validation, we compare the literature results with data from FDA approval. We assume combinations of tumor types and drugs that are approved by the FDA to be often reported in the literature and thus, to result in a significant association. And indeed, the vast majority of the combinations that are approved by the FDA shows a very significant support from the literature. 85% of the approved combinations show very high support from the literature (p-value $\le 10^{-100}$ for the overlap of publications). The other way around, almost 50% of the combinations of drugs and compounds with very high literature support are approved by the FDA.

**Fig. 2:**
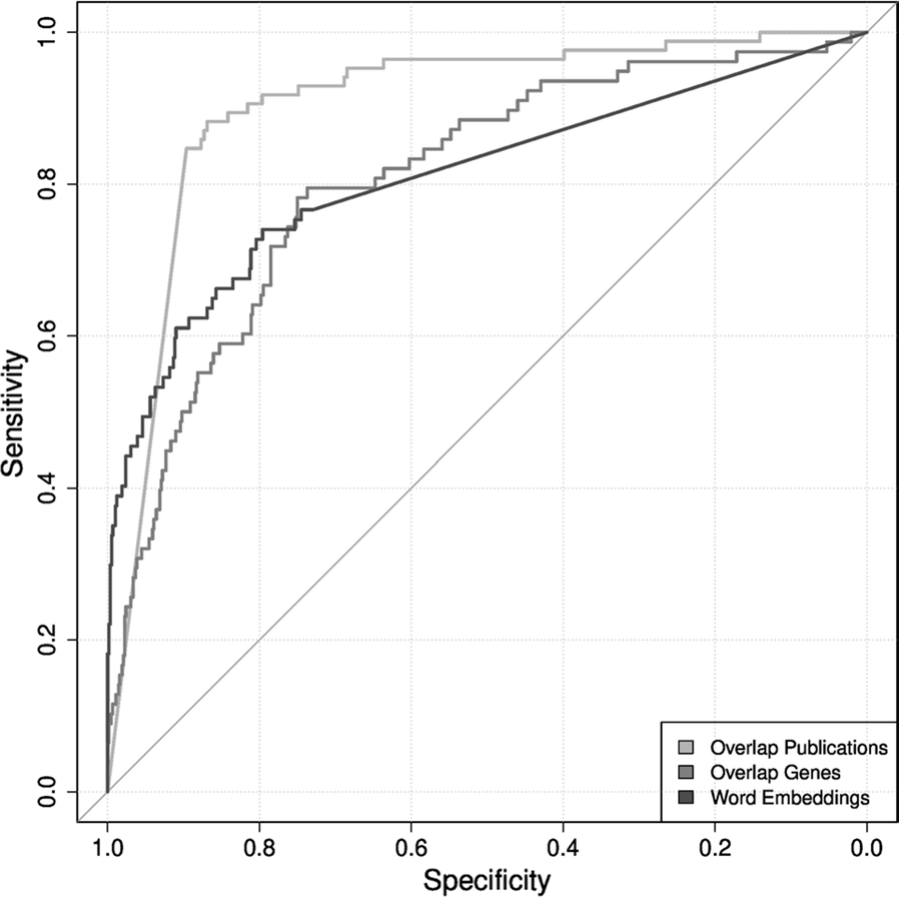
ROC curves FDA approval. Single value ROC curves comparing the scores ($\text{log}_{10}$p-value or cosine similarity) from the three approaches and the FDA approval data (prediction of approval)

In order to compare the three methods we calculated a single value ROC curve for a potential prediction of the FDA approval. Considering the overlap of publications, we receive an AUC of $\sim 0.9$ for the prediction of FDA approval by literature results (see Fig. 2). For the other two methods (overlap of genes and word embeddings) AUCs are $\sim 0.8$.

Only a very few FDA-approved combinations of drug and cancer-types show a low support from literature, e.g. imatinib and stomach cancer (We found only 47 publication for this combination while there are more than 8000 for imatinib and leukemia). When looking at the corresponding IC-50 values for stomach cancer cell lines we also see rather low support for this combination (lowest log IC-50 is 2 for imatinib and cell line SNU-1).

See Additional file 4: Figure S3 for a heatmap limited to the entities where we have information about FDA approval with highlighted entries.

#### IC-50 values

**Fig. 3:**
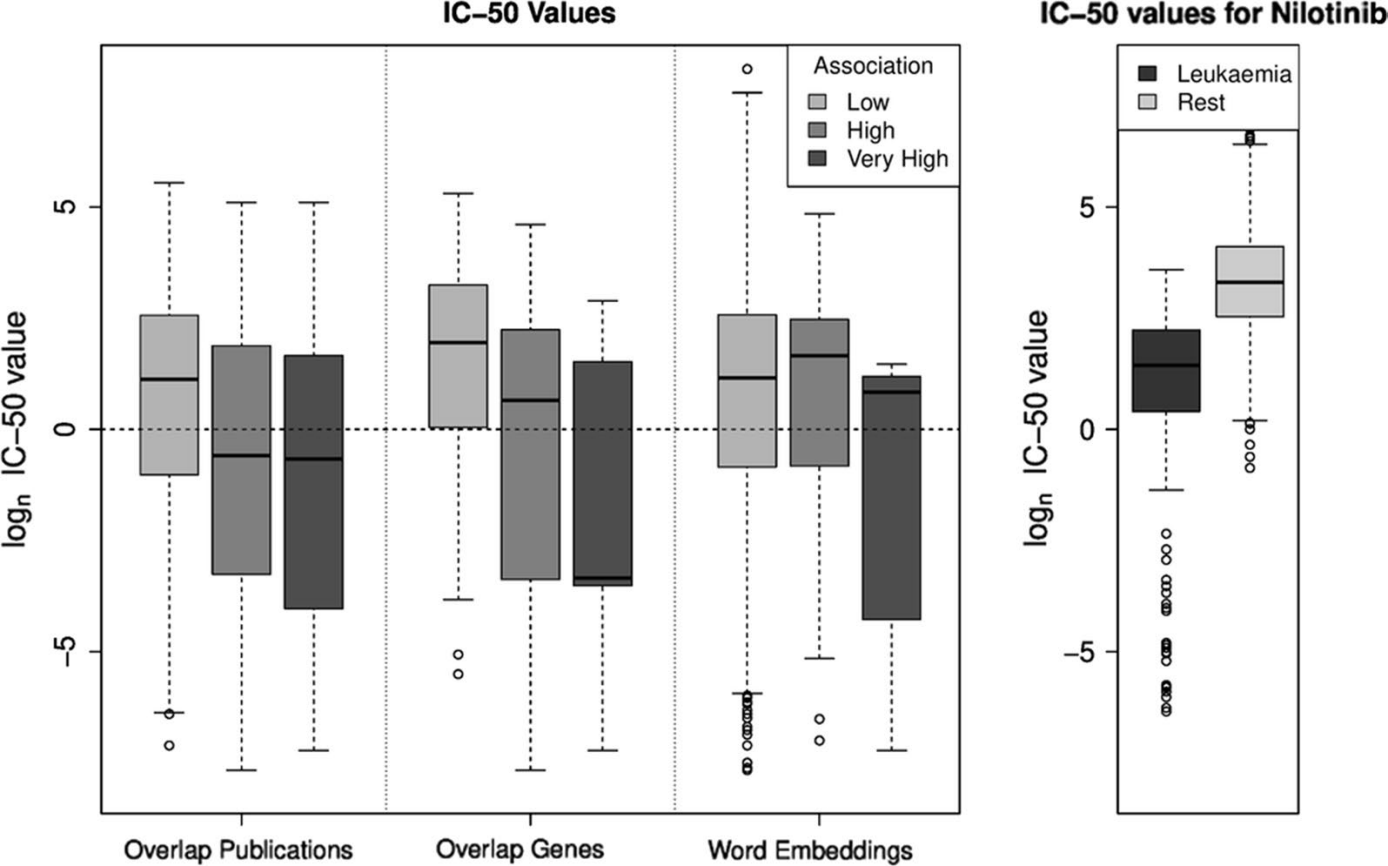
Valication with IC-50 values. Left hand side: boxplot of the IC-50 values for cancer/compound combinations with low, high and very high association. For ’Overlap Publications’ and ’Overlap Genes’ grouping is based on $-\text{log}_{10}$ Fisher test p-values (low: $<1$, high: between 20 and 80 and very high $\ge 80$). For ‘Word Embedding’ grouping is based on cosine similarity (low: $<0.1$, high: between 0.2 and 0.6 and very high $\ge 0.6$) Right hand side: boxplot of the IC-50 values for compound Nilotinib comparing leukemia derived cell lines with the remaining cell lines

As a second validation approach, we compare the significances of the relations between tumor types and compounds from literature analysis with the experimentally determined IC-50 values. We assume that a significant relation between a tumor type and a compound obtained by the literature data reflects a higher sensitivity of the tumor type to the compound and thus shows a lower IC-50 value. Especially for the first two methods (overlap of publications or genes), there is a high difference between the IC-50 values: IC-50 values for pairs of compounds and tumor types with a low association (p-value $> 0.1$) are $\sim$ 4-6 fold higher compared to the group with high association (p-value between $10^{-20}$ and $10^{-80}$) (see Fig. 3 for a boxplot of the IC-50 values). When considering the overlap of publications there is no further difference of the IC-50 value between a high (p-value between $10^{-20}$ and $10^{-80}$) and a very high association (p-value $\le 10^{-80}$). Considering significances of the relation based on overlap of genes, the difference of the IC-50 values is even higher comparing a high versus a very high association.

For word embeddings there is no difference in IC-50 values for cancer/compound combinations between low (cosine similarity $< 0.1$) and high cosine similarities (cosine similarity between 0.3 and 0.6). However, pairs of compounds and tumor types with very high cosine similarities ($> 0.6$) show slightly lower IC-50 values.

The observation that nilotinib is only related to leukemia can also be confirmed using the IC-50 values (see right hand side of Fig. 3). IC-50 values of leukemia cell lines treated with nilotinib are much lower ($\sim$ factor 6.5) compared to other cell lines.

#### Comparison with patient survival data

**Fig. 4:**
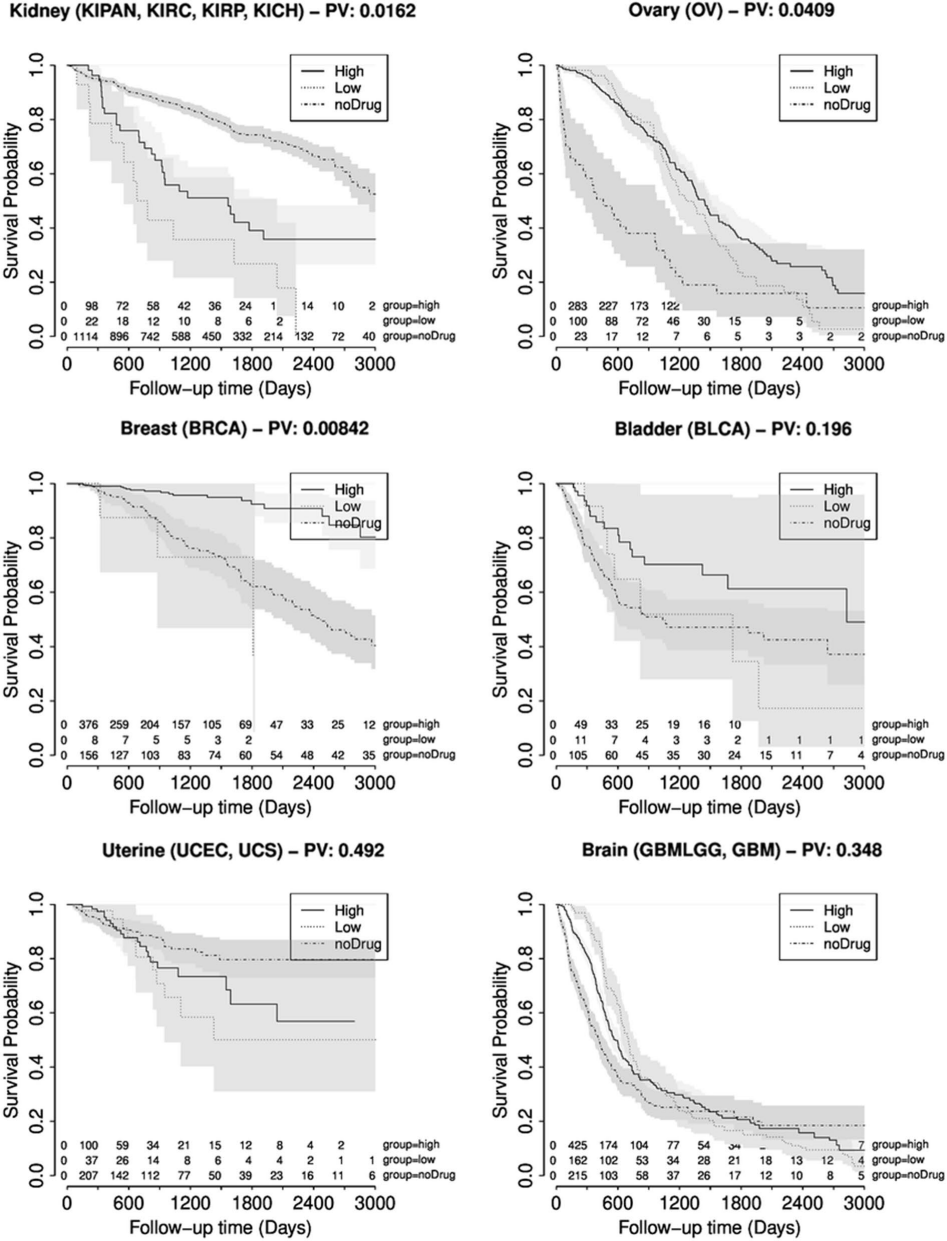
Kaplan–Meier survival curve. Kaplan–Meier survival curve for 6 selected tumor types (Top left: Kidney cancer, Top right: Ovary cancer, Middle left: Breast cancer, Middle right: Bladder cancer, Bottom left: Uterine cancer and Buttom right: Brain cancer). Three different survival curves are shown: noDrug: no drug was annotated for the patient, high: drugs with high literature support were annotated for the patient, low: drugs with low literature support were annotated for the patient. The p-value in the title compares the high vs. low group

As a third independent validation strategies the compare the significance of the relations from the literature mining with patient survival data. To this end, clinical patient data was parsed for the annotation of survival and treatment data (see Methods). The treatment data was mapped to the used compounds and three survival curves were generated: first, patients without drug annotation, second, patients that received drugs with high literature support and third, patients that received drugs with low literature support (see Fig. 4 for survival curves for kidney cancer, ovary cancer, breast cancer, bladder cancer, uterine cancer and brain cancer). Please note that for many cancer types we could not generate these survival curves since we found either only a very limited number of patients with clinical or exclusively drugs with high literature support. Due to the limited number of data we restrict the analysis to the approach with overlap of publications.

For the majority of cancer types where we found patients for all groups we observed lower survival rates for patients which received drugs with low literature support compared to patients receiving drugs with high literature support. For kidney cancer (0.016), lung cancer (0.008) and ovary cancer (0.04) we found significant differences between the corresponding survival curves. Patients with brain cancer show a slightly higher survival rate for drugs with low literature support at least for the first 900 days. For long term survival (> 900 days) we again observed an advantage for patients treated with drugs with high literature support.

### Accessibility of results in an interactive knowledge base

The interactive knowledge base is accessible freely using the following link:

http://www.compoundCancerKnowledgebase.com.

This resource can be used to perform in depth mining analyses using the data from this analysis. The user can select a compound and a cancer type either from a heatmap similar to Figure 1 or using an auto-completion search field. The results contain the following information:

1. A list of publications for the selected tumor and the selected cancer type. Occurrences of cancers, compounds and genes are highlighted for intuitive extraction of important information. Publications are linked to PubMed website.
2. A list of genes extracted from the set of corresponding publications. The genes are ordered according to absolute frequencies. The underlying publications can be accessed for further inspection. The gene symbols are visualized in a word cloud.
3. A list of protein protein interactions extracted from the set of corresponding publications. The interactions are visualized in an interactions graph using ‘Cytoscape.js’ [25] (Cytoscape, RRID:SCR_003032).
4. A list of the corresponding IC-50 scores from the cell lines of the selected tumor type and the selected compound.
5. A list of words with the highest similarities to the selected tumor type and the selected compound retrieved from word embeddings.
6. A list of co-occuring compounds and the co-occurrence graph for the corresponding tumor type.

All lists can be downloaded for further analyses in text based format.

For example, clicking on the leftmost upper cell (5’-Flourouracil and Leukaemia) retrieves 1320 publications having this co-occurrence. The tab ‘Genes’ refers to 288 cancer genes that have been found in these publications. The tab ‘Interactions’ gives us one interaction of proteins that has been reported. Further tabs show to compounds sensitivity values, word lists and related compounds.

## Discussion

By applying a large scale literature mining, we characterized the relation between 270 anti-cancer drugs and the 30 most frequent cancer types. The results are visualized in a large map depicting the relation between compounds, between cancer types as well as the mutual correspondence as reflected by the literature.

### Good agreement between literature and experimental data

The literature results were validated using three different approaches: first, by comparing with FDA approval data, second, based on experimentally measured IC-50 values and third, using clinical survival data. We observed a high agreement between the literature and the FDA drug approval. 85% of the approved combinations of drugs and cancer types showed very high support from the literature. The other way around, almost 50% of the combinations of drugs and compounds with very high literature support were approved by the FDA. The AUC of the ROC curve for a potential prediction of the FDA approval was high, up to $\sim 0.9$. Although this is a more or less expected result, it nicely shows the consistency of the results from literature mining with the clinical procedures and can be interpreted as a proof-of-principle.

In addition we were able to show that highly significant relations between a cancer type and a compound found in our literature mining analysis had lower experimentally measured IC-50 values compared to non-significant pairs of cancer types and compounds. Our analysis also showed that compounds specific for one cancer type, e.g. Nilotinib for leukemia, had low IC-50 values in these cell lines compared to cell lines for other cancer types. In other words, we have seen a good support of the literature results by experimental data.

The correspondence between the literature and experimental data might even be higher when including knowledge on molecular data such as genes or proteins. Comparing the association between cancer types and compounds based on the overlap of genes or proteins leads to a lower number of significant hits, but there is a strong support from experimental data. The lower number of significant results is mostly due to the lower number of publications, since less than 20% of the publications included genes or proteins.

The comparison of the results from literature mining with clinical survival data showed that drugs with higher literature support led to an improved survival compared to drugs with lower literature support for most tumor types. This comparison is limited by several factors: first, for some tumor types we did not find a sufficient number of patients with clinical data. Second, most of the applied drugs showed a high support from literature (see above: 85% of the approved drug/cancer type combinations showed very high support from the literature). Third, the application of non-approved drugs maybe the ‘last hope’ for the patient and thus these patients may have a very poor prognosis. Nevertheless, we see clear evidence that the literature support between drugs and cancer types correlates with the patient survival.

### General vs. specific compounds

We have seen that compounds can be divided in two groups: first, general compounds that are associated to many tumor types and second, specific compounds that are related to only one or two cancer types. This grouping corresponds to the mechanism of drug action. While general compounds typically target pathways that are not related to a specific tumor type but rather to growth and proliferation (‘DNA replication’ or ‘Mitosis’), specific compounds typically target signaling pathways that are important for one type of tumor. The grouping identified with the automated literature mining very nicely corresponds to the actual targets of the compound and thus, substantiates the consistency of the retrieved relations between tumor types and compounds.

### Identification of unexpected results

The heatmap showing the significances of the relations between cancer types and compounds (Fig. 1) can be used to identify unexpected results. As an example there is no significant relation between brain tumors and docetaxel, while all other tumor types in neighborhood as well as other compounds in the neighborhood show very significant results. So one would assume that docetaxel might be very useful to treat brain tumors. This assumption is underlined by experiments showing very low IC-50 values for different cell lines (data not shown here - but easily accessible in the knowledge base). This combination is even approved by the FDA (see also Additional file 4: Figure S3). The reason why there is almost no overlap between brain tumor and docetaxel are the physicochemical and pharmacological characteristics of the drug making the in vivo passage through blood-brain barrier extremely difficult [26].

### Drug repositioning

For each compound we searched for co-occuring compounds. This gives us for example for colon cancer 204 highly significant co-occuring pairs of drugs (with a co-occurrence $-\text{log}_{10}$ p-value $> 50$). This information can now be used for a proposition for drug repositioning. For example, mining for the EGFR targeting drug erlotinib reveals 224 related publications suggesting a high relevance of this drug for colon cancer ($-\text{log}_{10}$ p-value $= 77$). In contrast, afatinib, another EGFR-targeting drug, which has been used predominantly in lung cancer, has little literature relevance for colon cancer with only 37 publications ($-\text{log}_{10}$ p-value $= 2$). Nonetheless, afatinib appears as the top related drug with erlotinib from the combined information on colon cancer. This would suggest afatinib as a potential candidate for repositioning to colon cancer and there are indeed several phase 2 and 3 studies that propose afatinib for colon cancer therapy. This suggestion is in line with the IC-50 data. Many colon cancer cell lines are very sensitive to afatinib treatment (e.g. cell line DiFi with with log IC-50 values up to − 5.2).

### Drug combinations

The information of co-occuring compounds can also be used to search for promising drug combinations. E.g. the top co-occuring compound for 5-fluorouracil in colon cancer is the topoisomerase I inhibitor irinotecan (see also Additional file 5: Figure S4; all co-occurrrence graphs are provided as Additional file 6). 5-fluorouracil together with irinotecan and folinic acid (called FOLFIRI) were introduced as the standard of care for colorectal cancer (see [27] for a description of the drug-drug interactions).

As a second example, dabrafenib co-occurs with trametinib in different cancer types such as colon cancer or melanoma (see also Additional file 5: Figure S4). Combination therapy with dabrafenib and trametinib in melanoma improves response rate, progression-free survival and overall survival when compared to dabrafenib alone [28]. Both are targeting the ERK/MAPK signaling pathway, in particular the aberrant activation through BRAF mutations. The combination of dabrafenib and trametinib, targeting mitogen-activated extracellular signal-related kinase (MEK) which is downstream of BRAF in the MAPK pathway, was also approved by US FDA in 2014 based on increased survival over single dabrafenib monotherapy [29].

As a third example: the combination of bortezomib together with the immunomodulatory agents (IMiDs) such as lenalidomide has led to substantial improvement of survival rates in myeloma patients [30]. Both both compounds show a very significant co-occurrence.

### Comparison of the different approaches

We used two different text-mining approaches (classical text mining vs. word embeddings), both with a similar goal: the detection of similarity of cancer types and anti-cancer drugs based on scientific literature. The classical text mining is supervised since we directly included information about cancer types and compounds. The similarities were calculated only for cancer types and compounds incorporating the prior knowledge. Word embeddings are unsupervised. The abstracts were transformed into word vectors without considering knowledge about cancer types and compounds. The similarity is calculated based on the word vectors considering all possible other words. The supervised approach leads to a better correspondance with experimental data and seems to be better suited for the analysis task. Considering all possible words as in the word vector approach probably leads to a higher noise level and thus less significant results.

### Knowledge base for further data mining

We provide all results as an interactive web-based knowledge base. This knowledge base is freely accessible and can be used to further inspect the presented results. Researchers can use the knowledge base to follow their own research questions. Furthermore all data can be downloaded for performing additional analyses.

### Limitations

The presented analysis used techniques from literature mining to investigate the relation between compounds and tumor types. It is based on already published knowledge. Due to this reason it is per se not possible to discover completely new results. However, a combination of different aspects of published knowledge might be very helpful to discover new relations and to formulate novel hypotheses. We feel that especially the feature of co-occuring compounds could indeed be used to estimate efficacy of a drug in particular in the field of drug repositioning where on the one hand literature is available for the drug (a pre-requisite for every mining challenge) and on the other hand novel indication areas are to be investigated.

The restriction to abstracts instead of full-text publications has lower effects to the first part of the analysis (comparison of compounds and tumor types based on the overlap of publications) since the actual search is performed by PubMed search engine. However, the extraction of genes and proteins may be more precise when using full text publications. Also the word embeddings may benefit from using full text publications. Nevertheless, we decided to perform this analysis on abstracts especially since the many full text publications are not openly accessible and hence, the number of usable publications is significantly higher.

The description of the tumor types we used in our analysis is very general. E.g. breast cancer is not subdivided according to the status of estrogen-receptor, progesterone-receptor, HER2 or BRCA1. Although this sub-typing is very useful for the choice of treatment the vast majority of publication abstracts does not contain information on the tumor subtypes. Therefore we decided to use the high level of description of tumor types.

The body of 270 chemicals was motivated by the GDSC database [19] (RRID:SCR_011956). for which a rich body of data is available such as drug sensitivity information on a huge amount of human cell lines which is not generally available for every drug in drugbank. However, we plan to include more compounds in the next update of the knowledgebase tool.

## Conclusions

We demonstrated that an automated text mining is able to automatically assess the relations between the 30 most frequent cancer types and 270 anti-cancer drugs using biomedical publications (almost 4 million publications). To this end, we applied two different text mining strategies, first a supervised approach with classical text mining strategies and second an unsupervised approach based on the calculation of word embeddings. The supervised approach based on classical text mining seems to be better suited for the assessment of the relations cancer types and anti-cancer drugs. The relevance of the extracted relations was confirmed using three independent methods: First, by the comparison with FDA approval information, second, by using IC-50 values from a huge experimental dataset of compounds and cancer cell lines and third, by the comparison with clinical survival data. All three methods showed a good agreement between the results from automatic literature mining and independent validation.

In addition, we make the results of the analyses accessible in an interactive web-based knowledge base. The knowledge base enables researchers and clinicians to investigate relations between a certain combination of cancer type and compound very efficiently. All results can be exported for further in-depth analysis.

## Supplementary Information

**Additional file 1:**Zipped archive of the co-occurence graphs for all tumor types. Each co-occurrence graph contains all compounds in the context of the corresponding cancer type. Two compounds are connected if they show a highly significant co-occurrence with a − log_10_ p-value $> 50$. The color reflects the target pathway of the compound (extracted from the GDSC database (RRID:SCR_011956)).

**Additional file 2: Figure S1.** Heatmap visualizing the significance of the overlap in genes extracted from publications for compounds (y-axis) and cancer types (x-axis). The overlap is assessed based on the number of common genes extracted from publications in comparison to the total number of genes ($-\text{log}_{10}$ p-value of Fisher’s test truncated at 100). Compounds showing no significant association to anyof the tumor types are not shown.

**Additional file 3: Figure S2.** Heatmap visualizing the cosine similarity from word embedding between compounds (y-axis) and cancer types (x-axis).

**Additional file 4: Figure S3.** Heatmap visualizing the significance of the overlap between compounds (y-axis) and cancer types (x-axis). The overlap is assessed based on the number of common publications in comparison to the total number of publications ($-\text{log}_{10}$ p-value of Fisher’s test truncated at 100). We selected only the compounds and cancer types with information about approval by the FDA. If the combination of compound is approved for a cancer type than a blue box is drawn.

**Additional file 5: Figure S4.** Co-occurrence graph for all compounds in the context of colon cancer. Two compounds are connected if they show a highly significant co-occurrence with a − log_10_ p-value $> 50$. The color reflects the target pathway of the compound (extracted from the GDSC database (RRID:SCR_011956)).

**Additional file 6:** Text file with cancer type synonyms used for the literature search.

## Data Availability

All datasets used for this manuscript are publicly available. The sources are given in Methods section. All results are made accessible in an interactive web-based knowledge base using the following link: https://knowledgebase.microdiscovery.de/heatmap.

## Footnotes

1 https://www.cancer.gov/about-cancer/treatment/drugs.

2 https://www.wcrf.org/dietandcancer/cancer-trends/worldwide-cancer-data.
