# Supplementary figures and images for: Large-scale literature mining to assess the relation between anti-cancer drugs and cancer types
Source: J Transl Med. 2021 Jun 26;19:274. doi: 10.1186/s12967-021-02941-z (PMC8236166; doi:10.1186/s12967-021-02941-z)

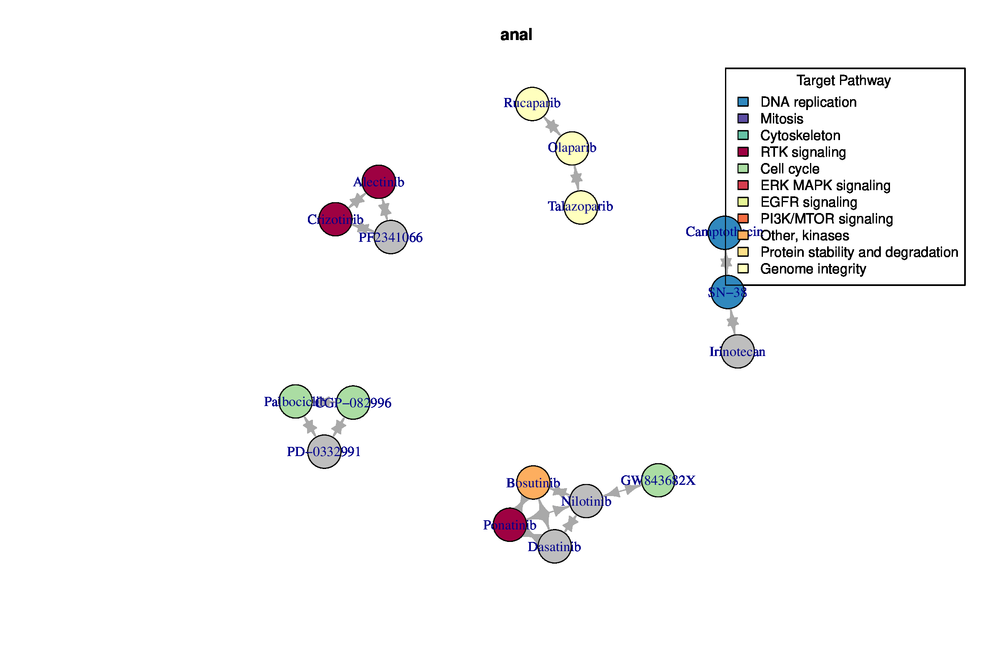

Supplement: Supplementary file 1 — Additional file 1:Zipped archive of the co-occurence graphs for all tumor types. Each co-occurrence graph contains all compounds in the context of the corresponding cancer type. Two compounds are connected if they show a highly significant co-occurrence with a − log10 p-value $> 50$>50. The color reflects the target pathway of the compound (extracted from the GDSC database (RRID:SCR_011956)). [file 12967_2021_2941_MOESM1_ESM.zip › drugDrugNetwork_anal_.png]

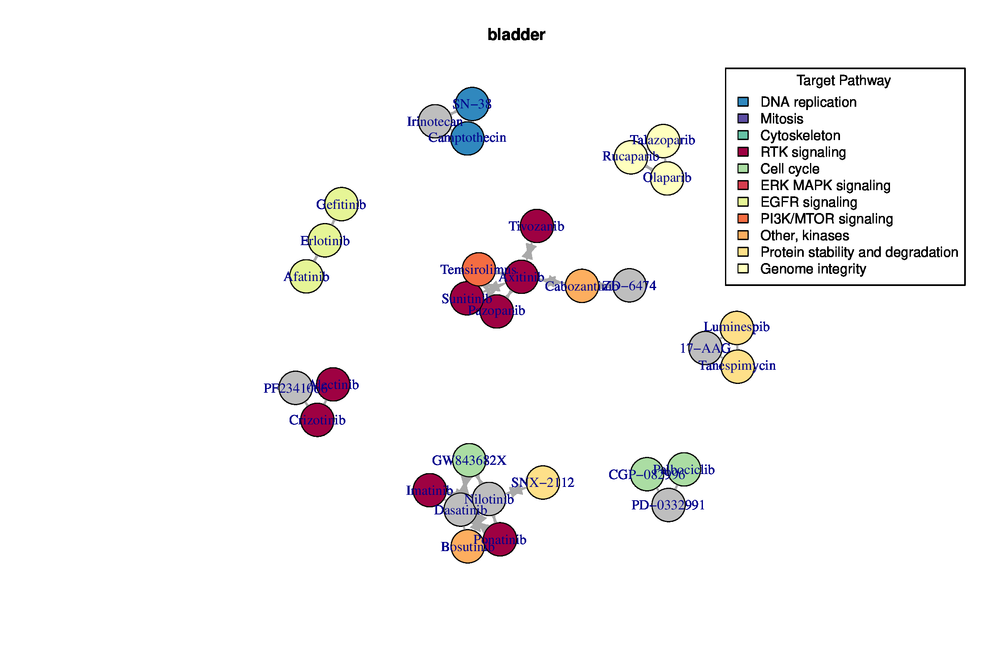

Supplement: Supplementary file 1 — Additional file 1:Zipped archive of the co-occurence graphs for all tumor types. Each co-occurrence graph contains all compounds in the context of the corresponding cancer type. Two compounds are connected if they show a highly significant co-occurrence with a − log10 p-value $> 50$>50. The color reflects the target pathway of the compound (extracted from the GDSC database (RRID:SCR_011956)). [file 12967_2021_2941_MOESM1_ESM.zip › drugDrugNetwork_bladder_.png]

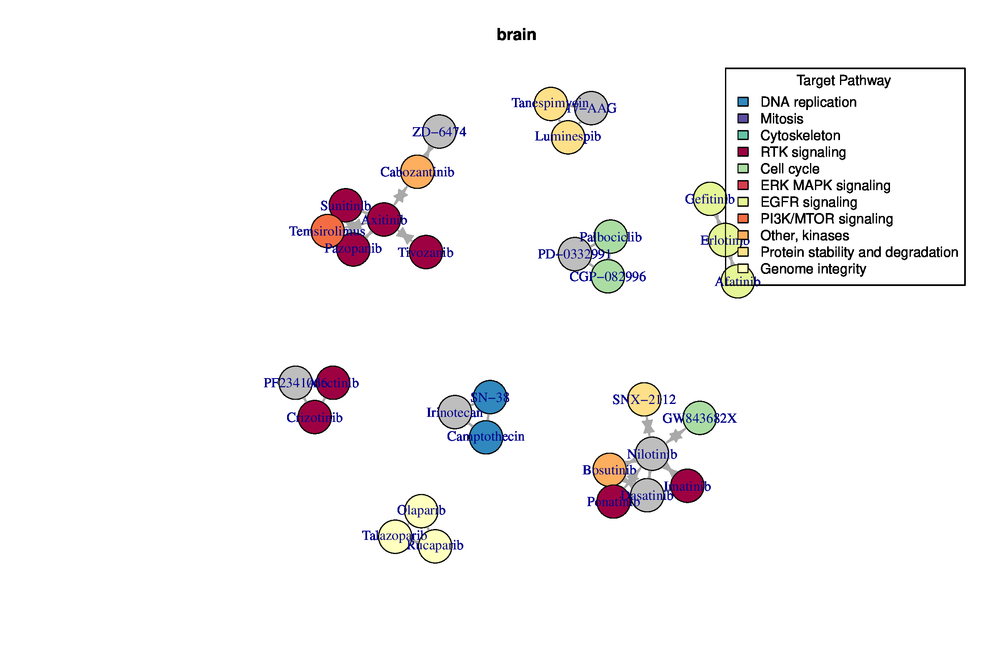

Supplement: Supplementary file 1 — Additional file 1:Zipped archive of the co-occurence graphs for all tumor types. Each co-occurrence graph contains all compounds in the context of the corresponding cancer type. Two compounds are connected if they show a highly significant co-occurrence with a − log10 p-value $> 50$>50. The color reflects the target pathway of the compound (extracted from the GDSC database (RRID:SCR_011956)). [file 12967_2021_2941_MOESM1_ESM.zip › drugDrugNetwork_brain_.png]

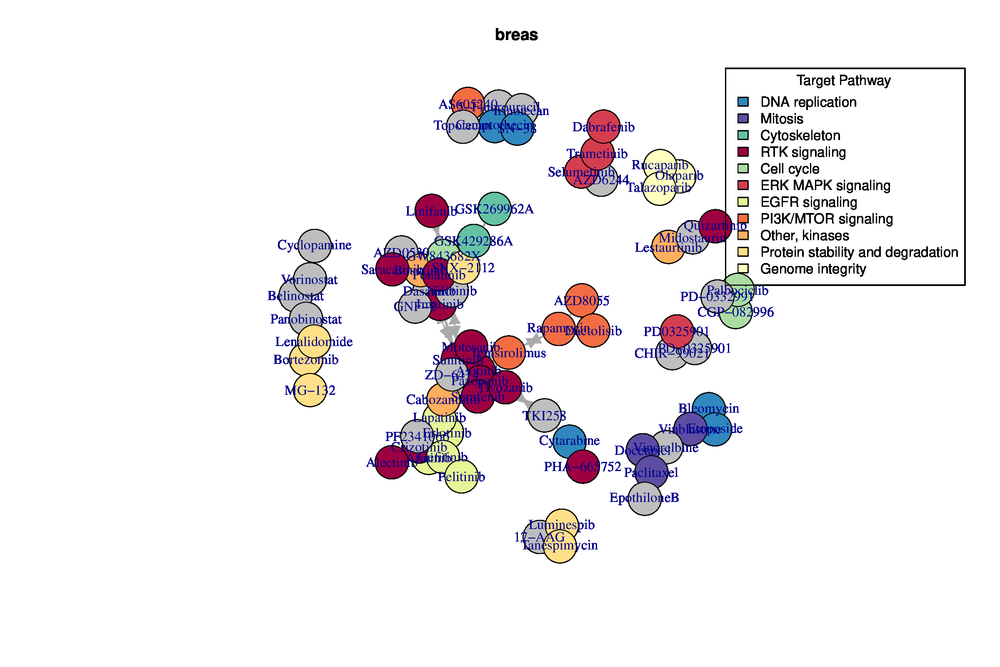

Supplement: Supplementary file 1 — Additional file 1:Zipped archive of the co-occurence graphs for all tumor types. Each co-occurrence graph contains all compounds in the context of the corresponding cancer type. Two compounds are connected if they show a highly significant co-occurrence with a − log10 p-value $> 50$>50. The color reflects the target pathway of the compound (extracted from the GDSC database (RRID:SCR_011956)). [file 12967_2021_2941_MOESM1_ESM.zip › drugDrugNetwork_breas_.png]

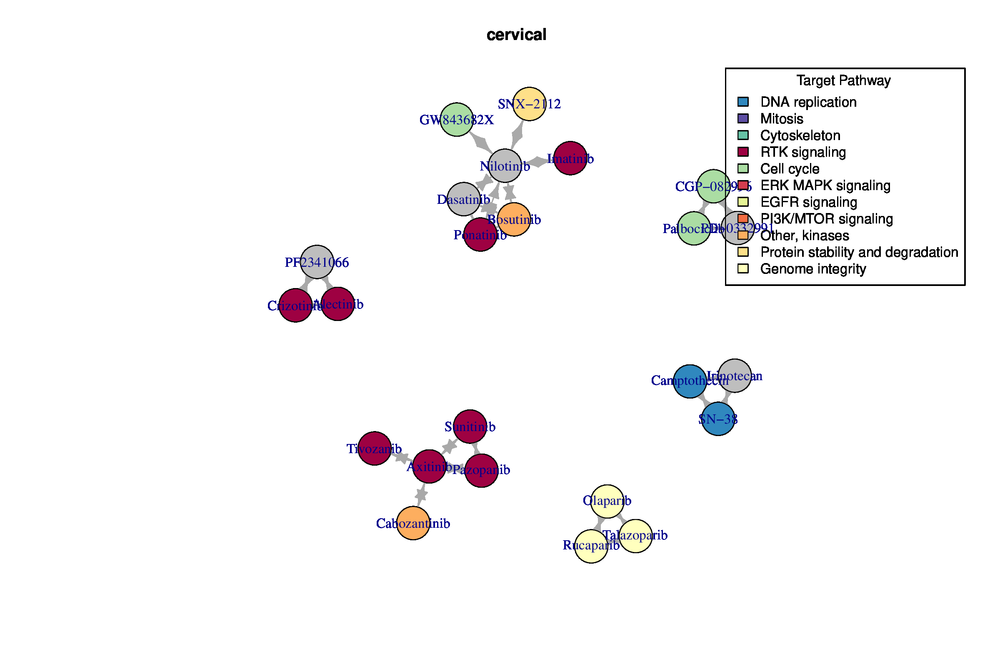

Supplement: Supplementary file 1 — Additional file 1:Zipped archive of the co-occurence graphs for all tumor types. Each co-occurrence graph contains all compounds in the context of the corresponding cancer type. Two compounds are connected if they show a highly significant co-occurrence with a − log10 p-value $> 50$>50. The color reflects the target pathway of the compound (extracted from the GDSC database (RRID:SCR_011956)). [file 12967_2021_2941_MOESM1_ESM.zip › drugDrugNetwork_cervical_.png]

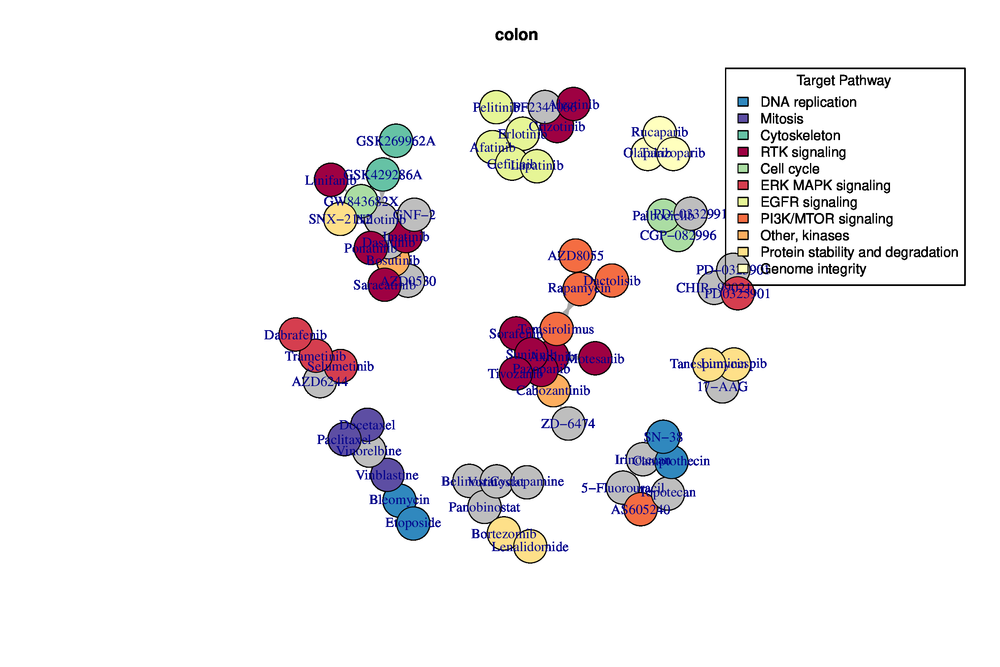

Supplement: Supplementary file 1 — Additional file 1:Zipped archive of the co-occurence graphs for all tumor types. Each co-occurrence graph contains all compounds in the context of the corresponding cancer type. Two compounds are connected if they show a highly significant co-occurrence with a − log10 p-value $> 50$>50. The color reflects the target pathway of the compound (extracted from the GDSC database (RRID:SCR_011956)). [file 12967_2021_2941_MOESM1_ESM.zip › drugDrugNetwork_colon_.png]

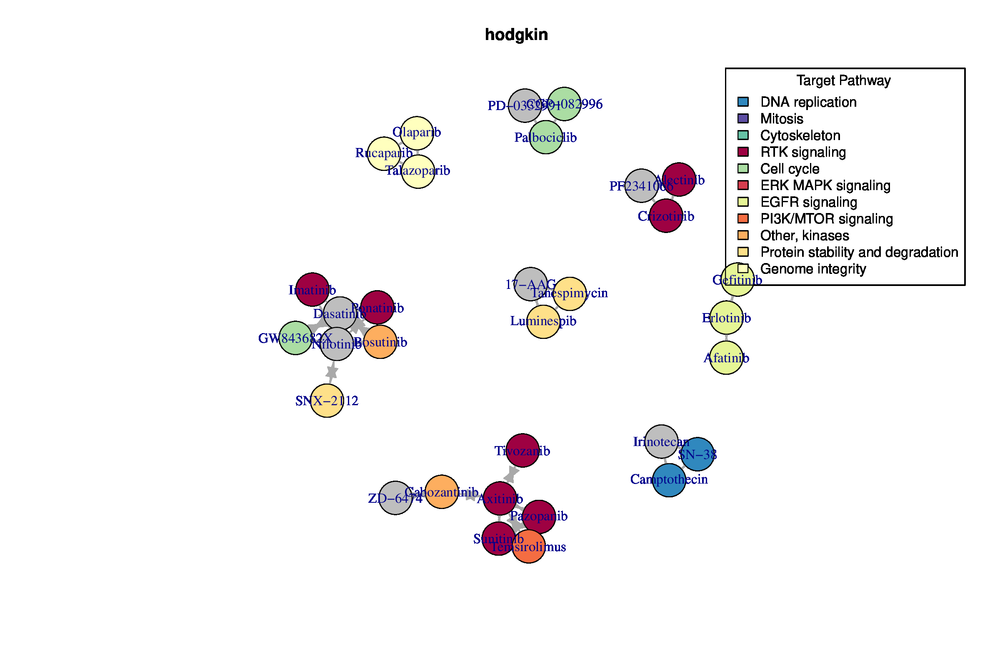

Supplement: Supplementary file 1 — Additional file 1:Zipped archive of the co-occurence graphs for all tumor types. Each co-occurrence graph contains all compounds in the context of the corresponding cancer type. Two compounds are connected if they show a highly significant co-occurrence with a − log10 p-value $> 50$>50. The color reflects the target pathway of the compound (extracted from the GDSC database (RRID:SCR_011956)). [file 12967_2021_2941_MOESM1_ESM.zip › drugDrugNetwork_hodgkin_.png]

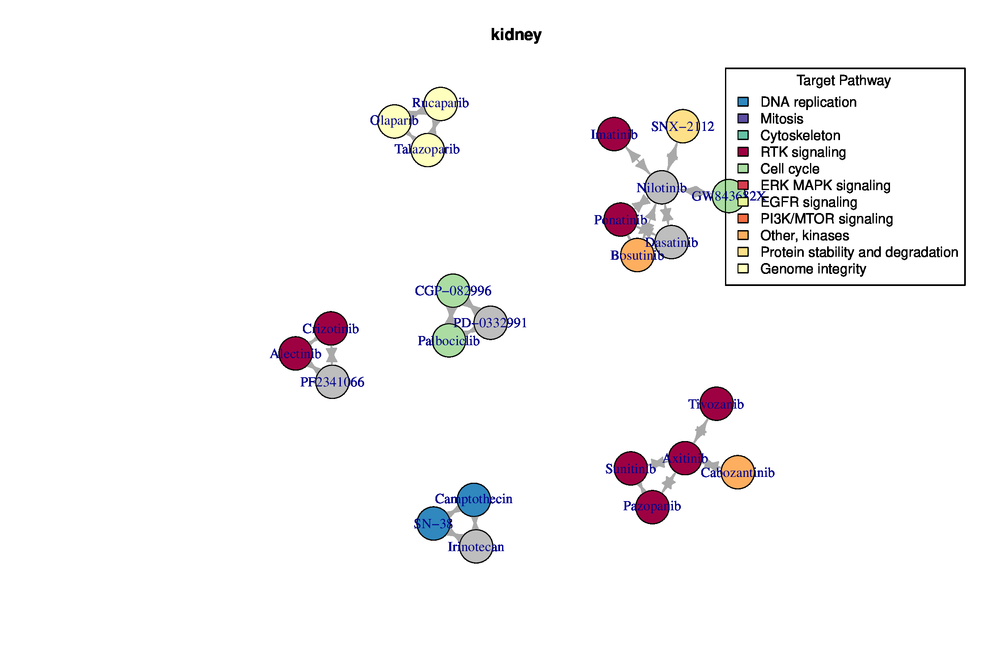

Supplement: Supplementary file 1 — Additional file 1:Zipped archive of the co-occurence graphs for all tumor types. Each co-occurrence graph contains all compounds in the context of the corresponding cancer type. Two compounds are connected if they show a highly significant co-occurrence with a − log10 p-value $> 50$>50. The color reflects the target pathway of the compound (extracted from the GDSC database (RRID:SCR_011956)). [file 12967_2021_2941_MOESM1_ESM.zip › drugDrugNetwork_kidney_.png]

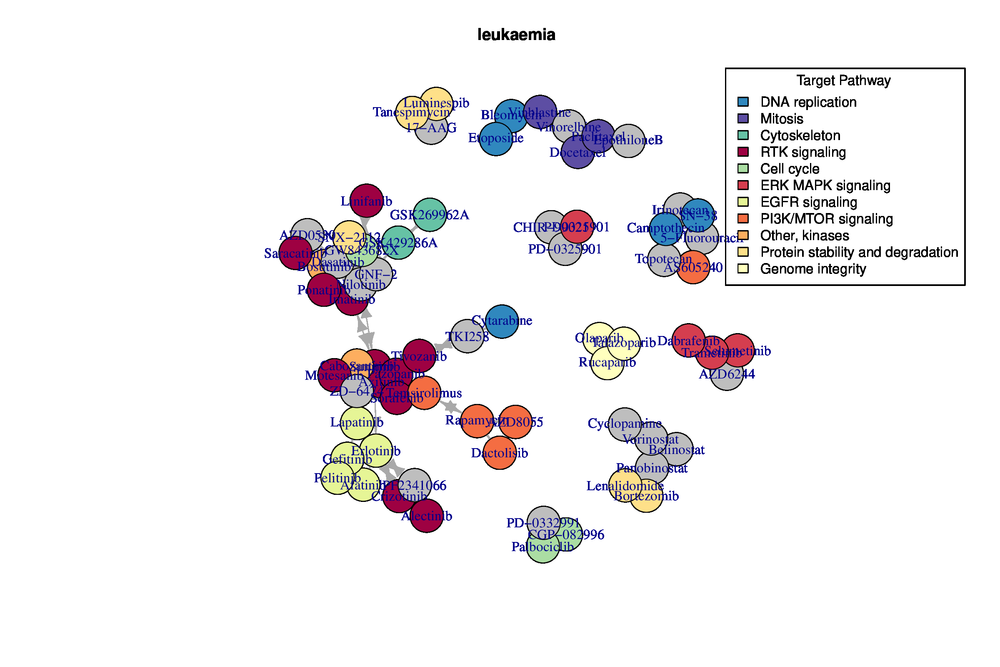

Supplement: Supplementary file 1 — Additional file 1:Zipped archive of the co-occurence graphs for all tumor types. Each co-occurrence graph contains all compounds in the context of the corresponding cancer type. Two compounds are connected if they show a highly significant co-occurrence with a − log10 p-value $> 50$>50. The color reflects the target pathway of the compound (extracted from the GDSC database (RRID:SCR_011956)). [file 12967_2021_2941_MOESM1_ESM.zip › drugDrugNetwork_leukaemia_.png]

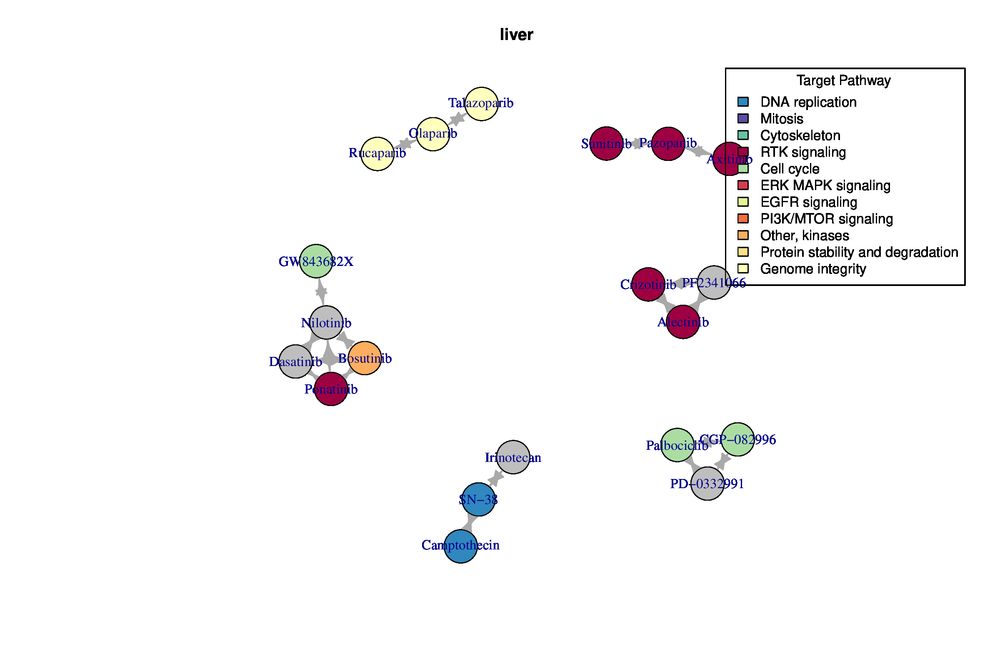

Supplement: Supplementary file 1 — Additional file 1:Zipped archive of the co-occurence graphs for all tumor types. Each co-occurrence graph contains all compounds in the context of the corresponding cancer type. Two compounds are connected if they show a highly significant co-occurrence with a − log10 p-value $> 50$>50. The color reflects the target pathway of the compound (extracted from the GDSC database (RRID:SCR_011956)). [file 12967_2021_2941_MOESM1_ESM.zip › drugDrugNetwork_liver_.png]

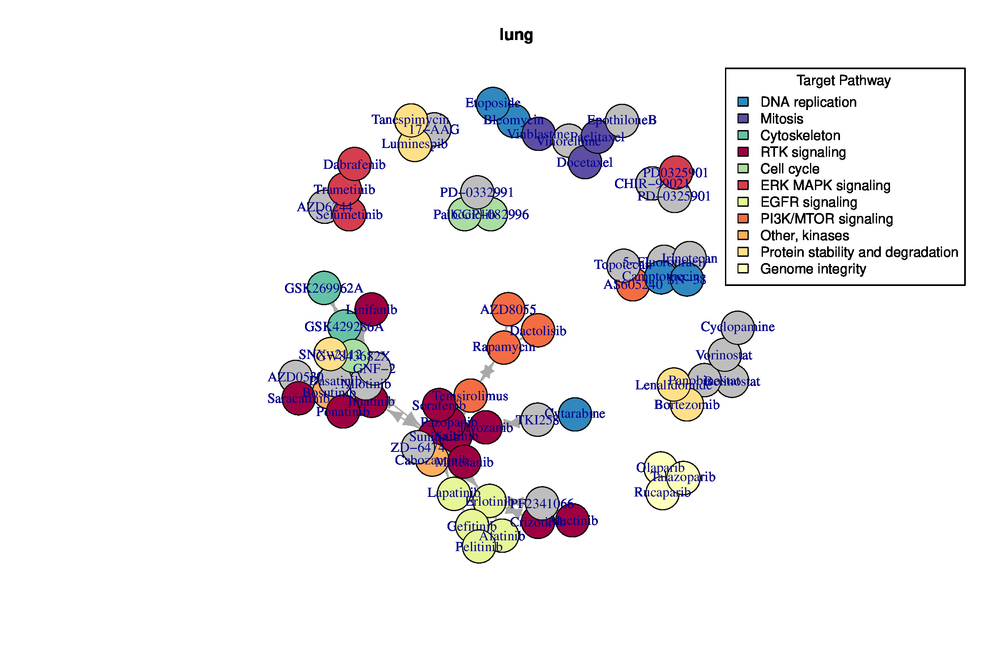

Supplement: Supplementary file 1 — Additional file 1:Zipped archive of the co-occurence graphs for all tumor types. Each co-occurrence graph contains all compounds in the context of the corresponding cancer type. Two compounds are connected if they show a highly significant co-occurrence with a − log10 p-value $> 50$>50. The color reflects the target pathway of the compound (extracted from the GDSC database (RRID:SCR_011956)). [file 12967_2021_2941_MOESM1_ESM.zip › drugDrugNetwork_lung_.png]

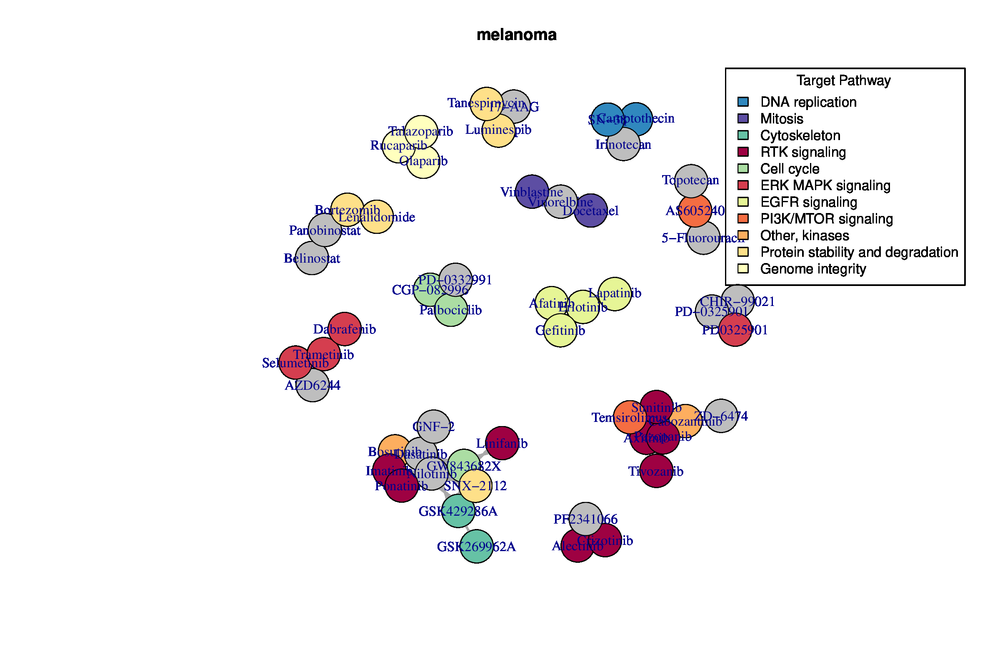

Supplement: Supplementary file 1 — Additional file 1:Zipped archive of the co-occurence graphs for all tumor types. Each co-occurrence graph contains all compounds in the context of the corresponding cancer type. Two compounds are connected if they show a highly significant co-occurrence with a − log10 p-value $> 50$>50. The color reflects the target pathway of the compound (extracted from the GDSC database (RRID:SCR_011956)). [file 12967_2021_2941_MOESM1_ESM.zip › drugDrugNetwork_melanoma_.png]

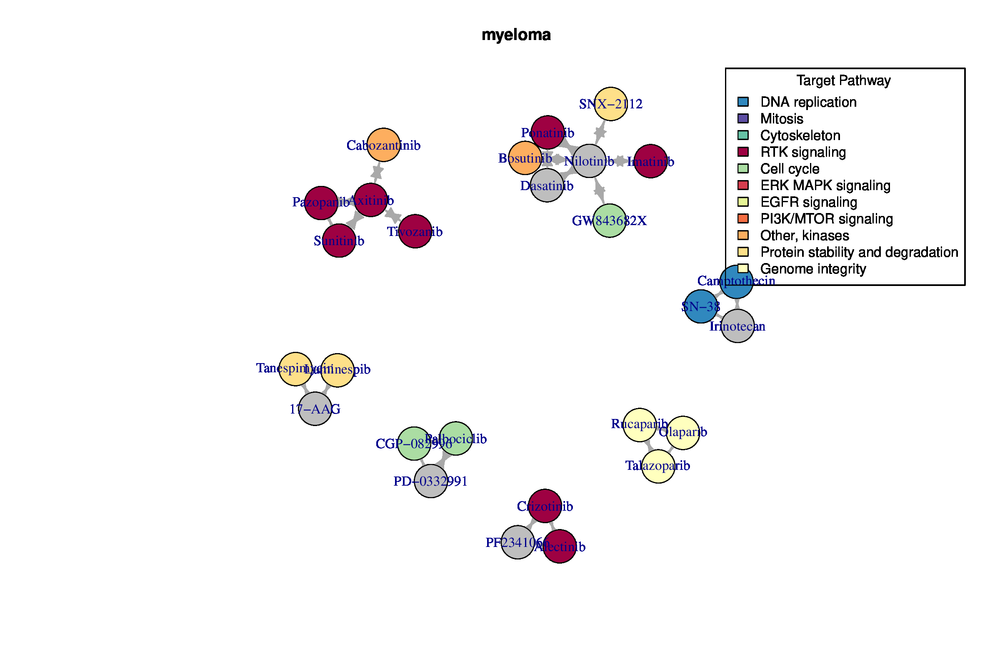

Supplement: Supplementary file 1 — Additional file 1:Zipped archive of the co-occurence graphs for all tumor types. Each co-occurrence graph contains all compounds in the context of the corresponding cancer type. Two compounds are connected if they show a highly significant co-occurrence with a − log10 p-value $> 50$>50. The color reflects the target pathway of the compound (extracted from the GDSC database (RRID:SCR_011956)). [file 12967_2021_2941_MOESM1_ESM.zip › drugDrugNetwork_myeloma_.png]

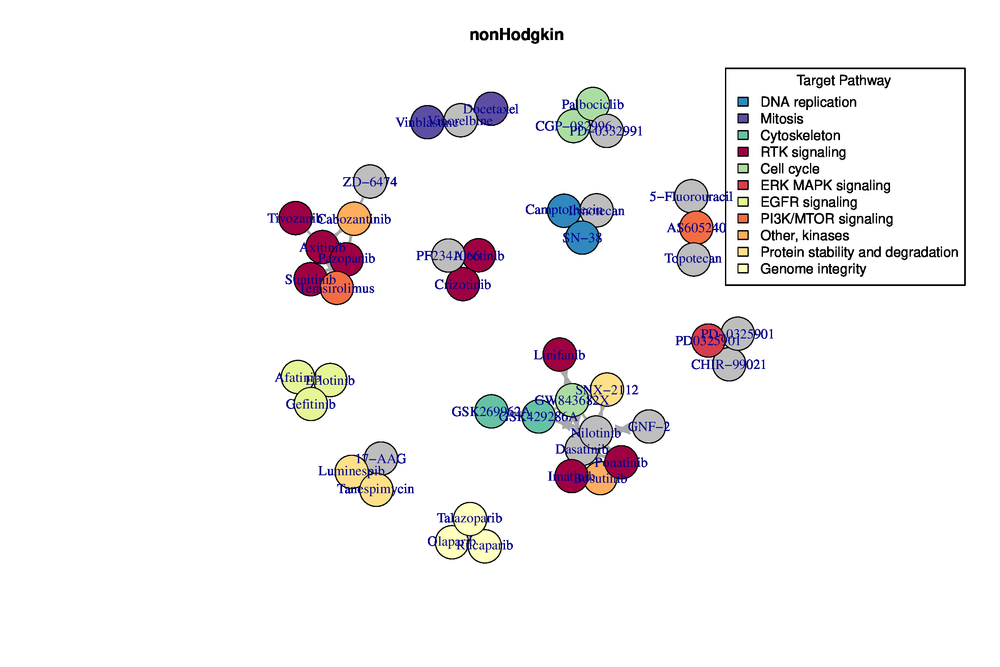

Supplement: Supplementary file 1 — Additional file 1:Zipped archive of the co-occurence graphs for all tumor types. Each co-occurrence graph contains all compounds in the context of the corresponding cancer type. Two compounds are connected if they show a highly significant co-occurrence with a − log10 p-value $> 50$>50. The color reflects the target pathway of the compound (extracted from the GDSC database (RRID:SCR_011956)). [file 12967_2021_2941_MOESM1_ESM.zip › drugDrugNetwork_nonHodgkin_.png]

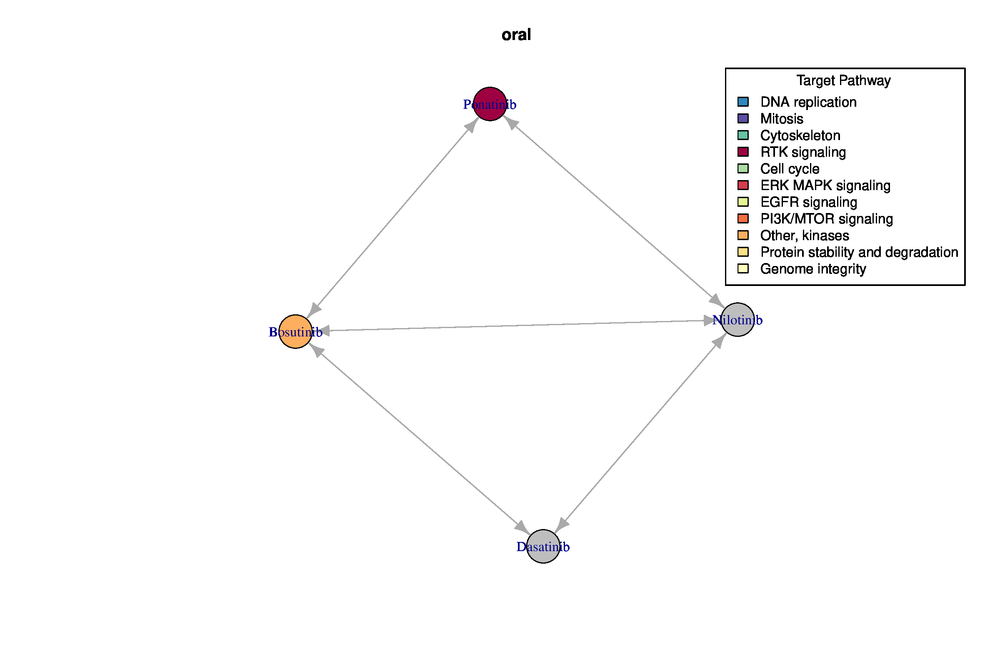

Supplement: Supplementary file 1 — Additional file 1:Zipped archive of the co-occurence graphs for all tumor types. Each co-occurrence graph contains all compounds in the context of the corresponding cancer type. Two compounds are connected if they show a highly significant co-occurrence with a − log10 p-value $> 50$>50. The color reflects the target pathway of the compound (extracted from the GDSC database (RRID:SCR_011956)). [file 12967_2021_2941_MOESM1_ESM.zip › drugDrugNetwork_oral_.png]

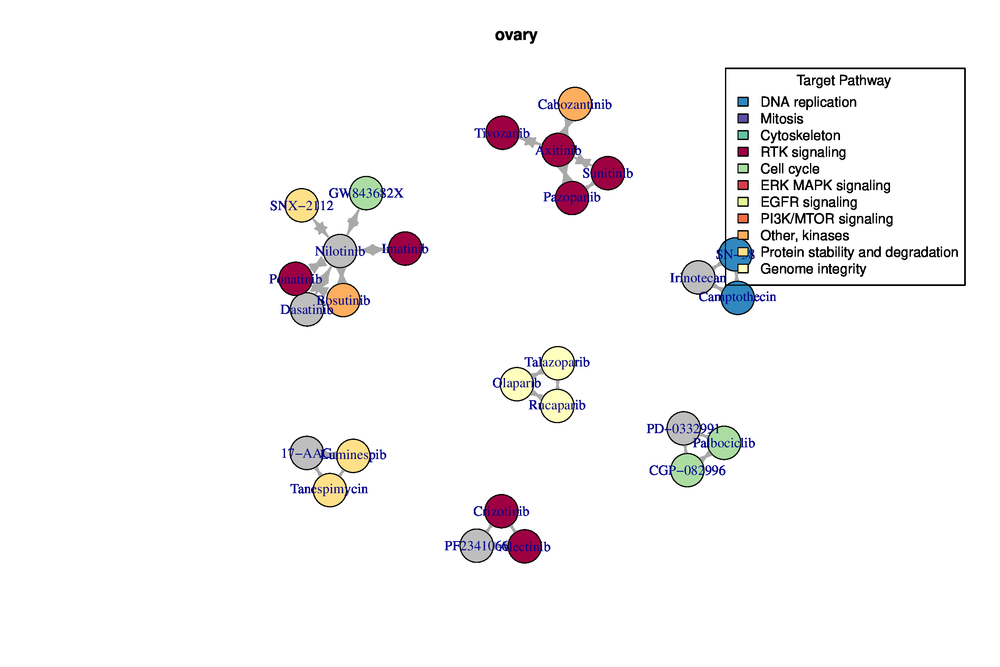

Supplement: Supplementary file 1 — Additional file 1:Zipped archive of the co-occurence graphs for all tumor types. Each co-occurrence graph contains all compounds in the context of the corresponding cancer type. Two compounds are connected if they show a highly significant co-occurrence with a − log10 p-value $> 50$>50. The color reflects the target pathway of the compound (extracted from the GDSC database (RRID:SCR_011956)). [file 12967_2021_2941_MOESM1_ESM.zip › drugDrugNetwork_ovary_.png]

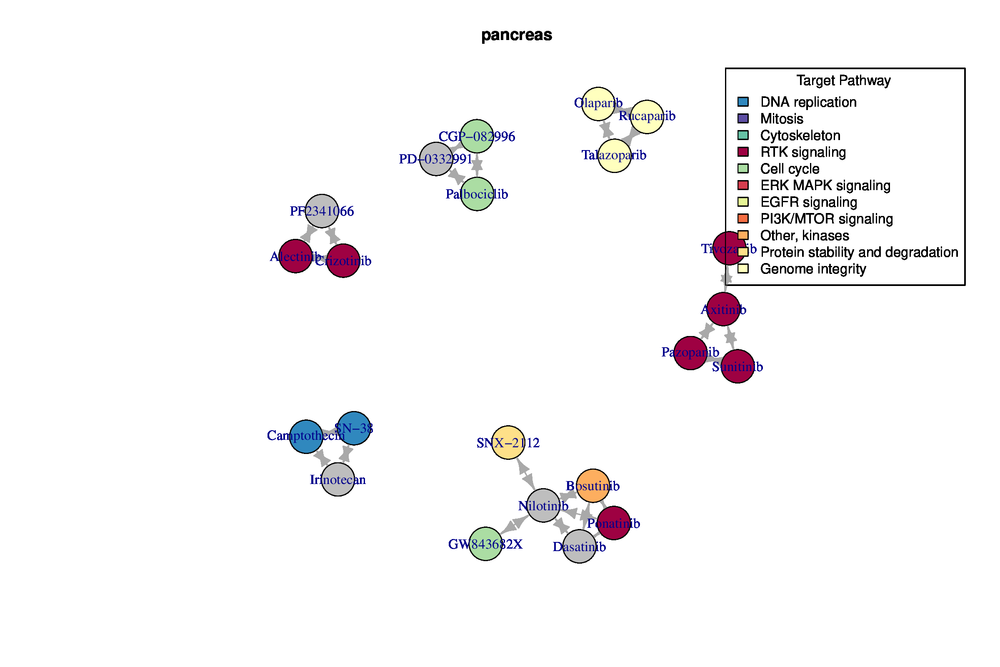

Supplement: Supplementary file 1 — Additional file 1:Zipped archive of the co-occurence graphs for all tumor types. Each co-occurrence graph contains all compounds in the context of the corresponding cancer type. Two compounds are connected if they show a highly significant co-occurrence with a − log10 p-value $> 50$>50. The color reflects the target pathway of the compound (extracted from the GDSC database (RRID:SCR_011956)). [file 12967_2021_2941_MOESM1_ESM.zip › drugDrugNetwork_pancreas_.png]

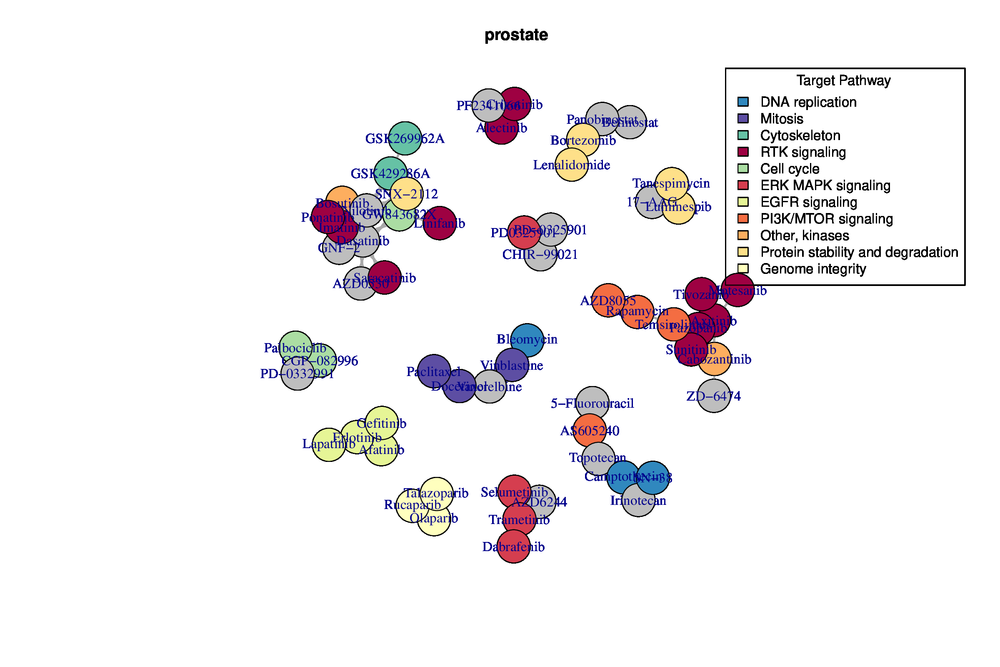

Supplement: Supplementary file 1 — Additional file 1:Zipped archive of the co-occurence graphs for all tumor types. Each co-occurrence graph contains all compounds in the context of the corresponding cancer type. Two compounds are connected if they show a highly significant co-occurrence with a − log10 p-value $> 50$>50. The color reflects the target pathway of the compound (extracted from the GDSC database (RRID:SCR_011956)). [file 12967_2021_2941_MOESM1_ESM.zip › drugDrugNetwork_prostate_.png]

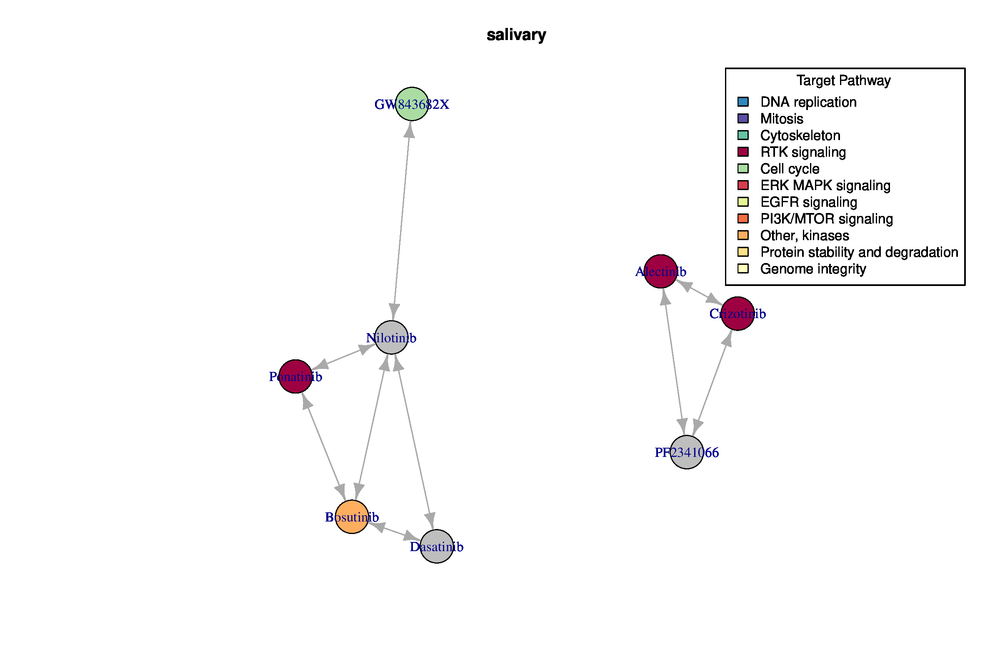

Supplement: Supplementary file 1 — Additional file 1:Zipped archive of the co-occurence graphs for all tumor types. Each co-occurrence graph contains all compounds in the context of the corresponding cancer type. Two compounds are connected if they show a highly significant co-occurrence with a − log10 p-value $> 50$>50. The color reflects the target pathway of the compound (extracted from the GDSC database (RRID:SCR_011956)). [file 12967_2021_2941_MOESM1_ESM.zip › drugDrugNetwork_salivary_.png]

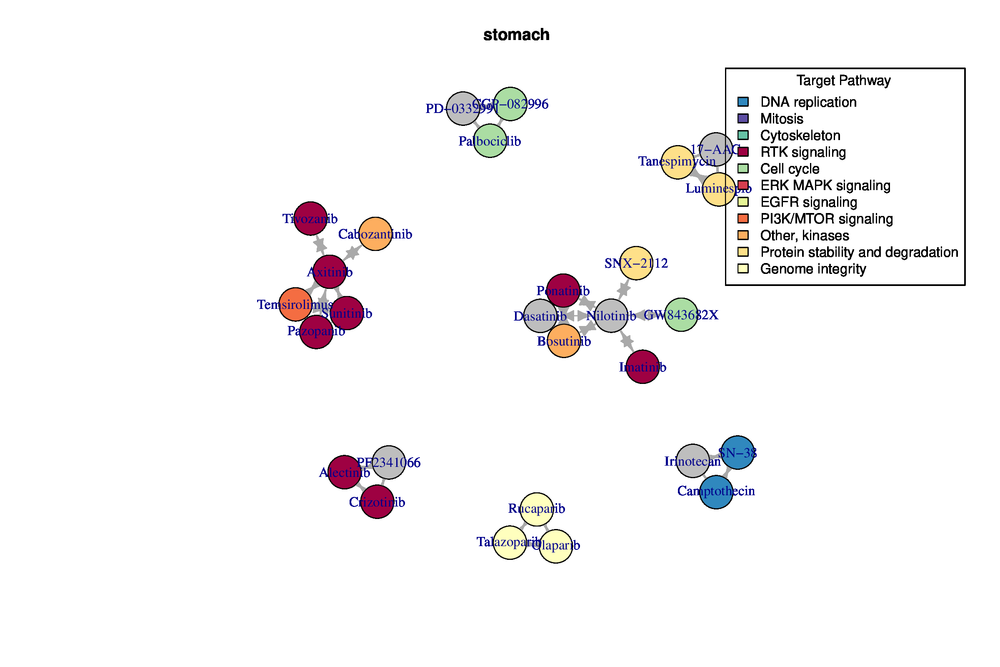

Supplement: Supplementary file 1 — Additional file 1:Zipped archive of the co-occurence graphs for all tumor types. Each co-occurrence graph contains all compounds in the context of the corresponding cancer type. Two compounds are connected if they show a highly significant co-occurrence with a − log10 p-value $> 50$>50. The color reflects the target pathway of the compound (extracted from the GDSC database (RRID:SCR_011956)). [file 12967_2021_2941_MOESM1_ESM.zip › drugDrugNetwork_stomach_.png]

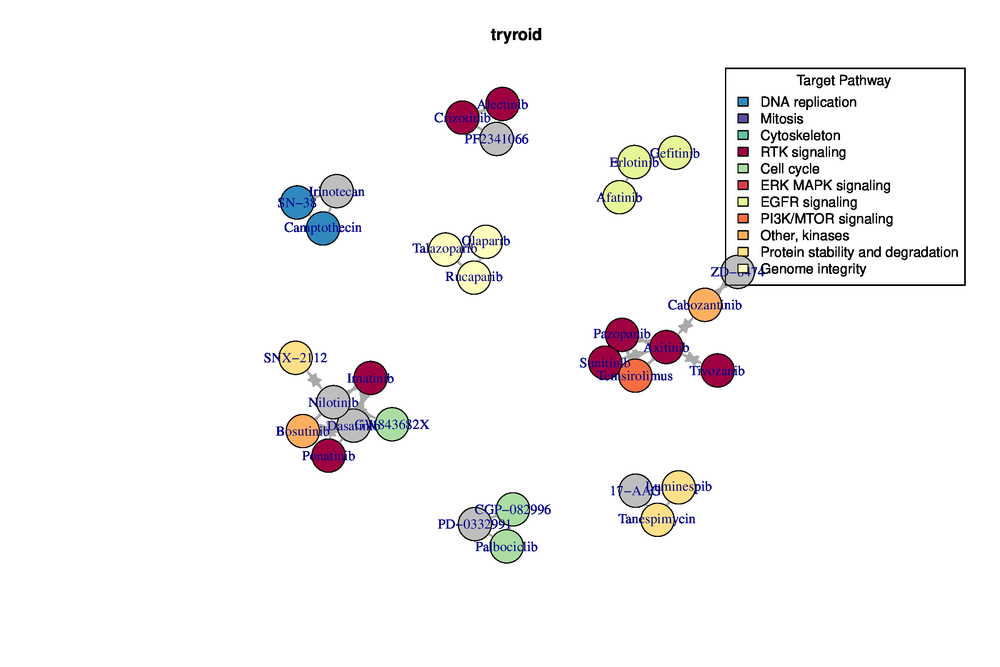

Supplement: Supplementary file 1 — Additional file 1:Zipped archive of the co-occurence graphs for all tumor types. Each co-occurrence graph contains all compounds in the context of the corresponding cancer type. Two compounds are connected if they show a highly significant co-occurrence with a − log10 p-value $> 50$>50. The color reflects the target pathway of the compound (extracted from the GDSC database (RRID:SCR_011956)). [file 12967_2021_2941_MOESM1_ESM.zip › drugDrugNetwork_tryroid_.png]

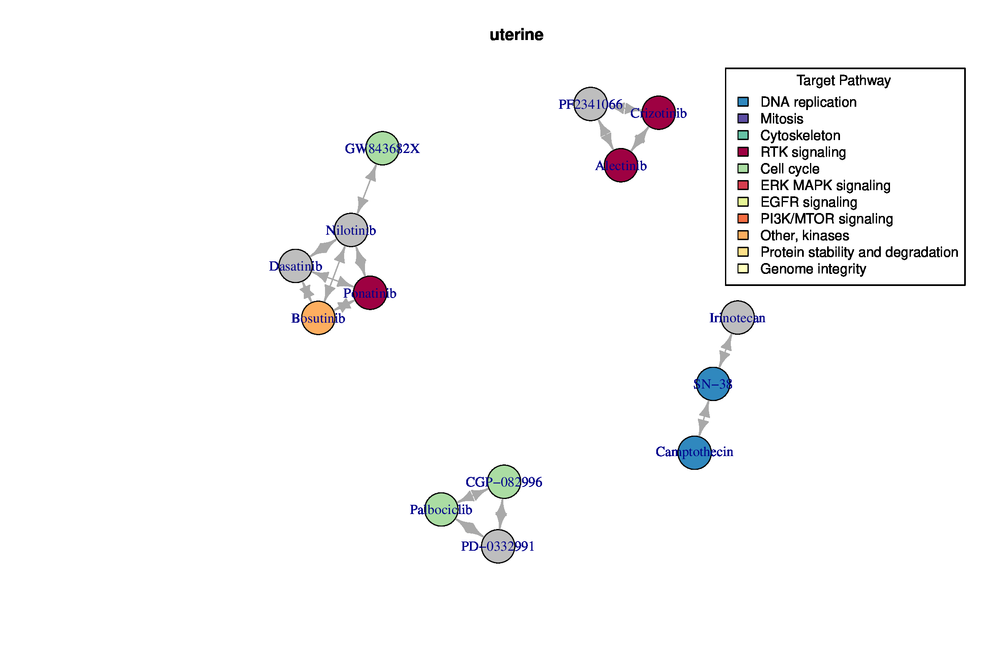

Supplement: Supplementary file 1 — Additional file 1:Zipped archive of the co-occurence graphs for all tumor types. Each co-occurrence graph contains all compounds in the context of the corresponding cancer type. Two compounds are connected if they show a highly significant co-occurrence with a − log10 p-value $> 50$>50. The color reflects the target pathway of the compound (extracted from the GDSC database (RRID:SCR_011956)). [file 12967_2021_2941_MOESM1_ESM.zip › drugDrugNetwork_uterine_.png]

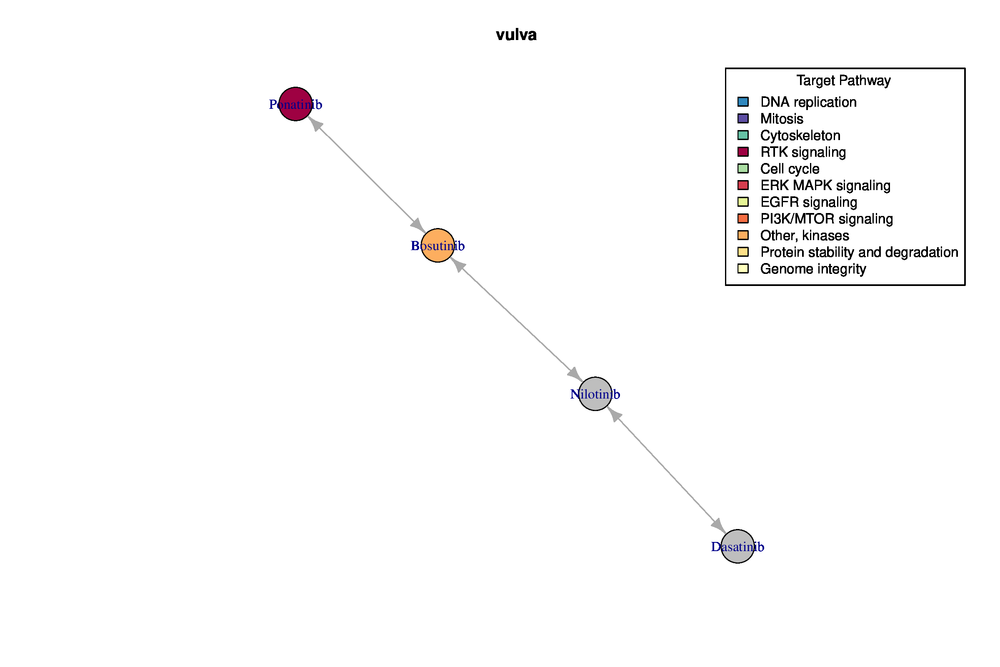

Supplement: Supplementary file 1 — Additional file 1:Zipped archive of the co-occurence graphs for all tumor types. Each co-occurrence graph contains all compounds in the context of the corresponding cancer type. Two compounds are connected if they show a highly significant co-occurrence with a − log10 p-value $> 50$>50. The color reflects the target pathway of the compound (extracted from the GDSC database (RRID:SCR_011956)). [file 12967_2021_2941_MOESM1_ESM.zip › drugDrugNetwork_vulva_.png]

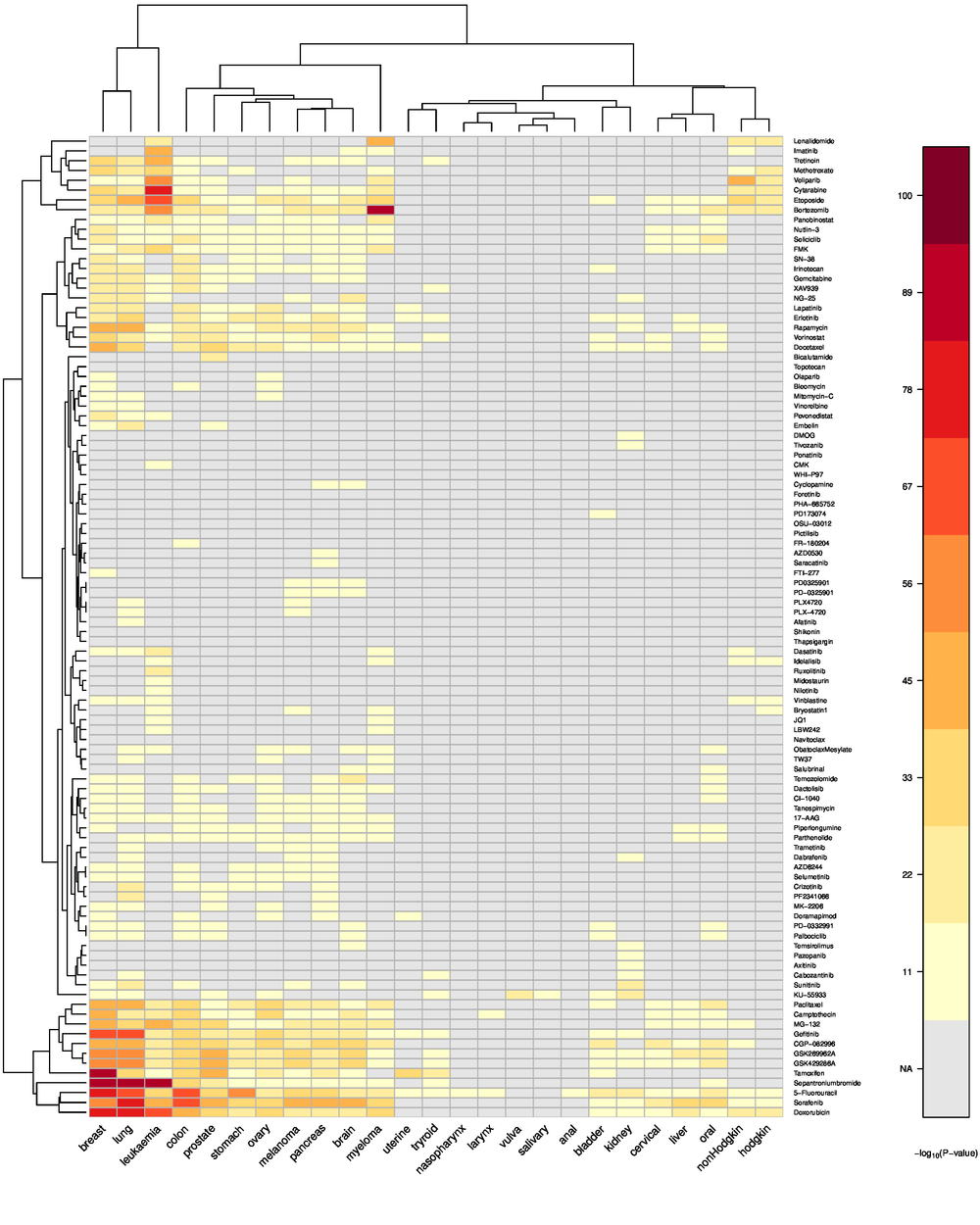

Supplement: Supplementary file 2 — Additional file 2: Figure S1. Heatmap visualizing the significance of the overlap in genes extracted from publications for compounds (y-axis) and cancer types (x-axis). The overlap is assessed based on the number of common genes extracted from publications in comparison to the total number of genes ($-\text{log}_{10}$-log10 p-value of Fisher’s test truncated at 100). Compounds showing no significant association to anyof the tumor types are not shown. [file 12967_2021_2941_MOESM2_ESM.png]

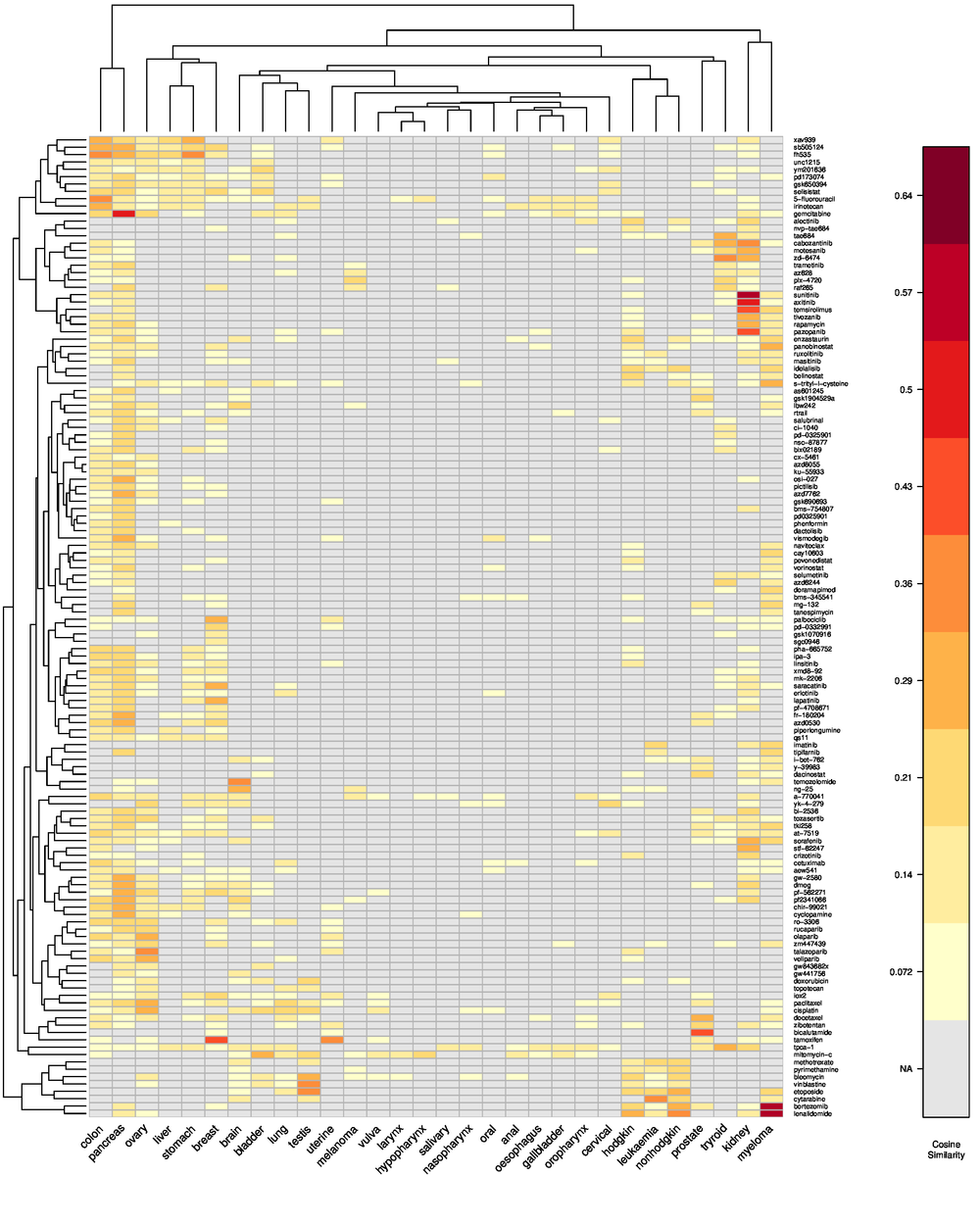

Supplement: Supplementary file 3 — Additional file 3: Figure S2. Heatmap visualizing the cosine similarity from word embedding between compounds (y-axis) and cancer types (x-axis). [file 12967_2021_2941_MOESM3_ESM.png]

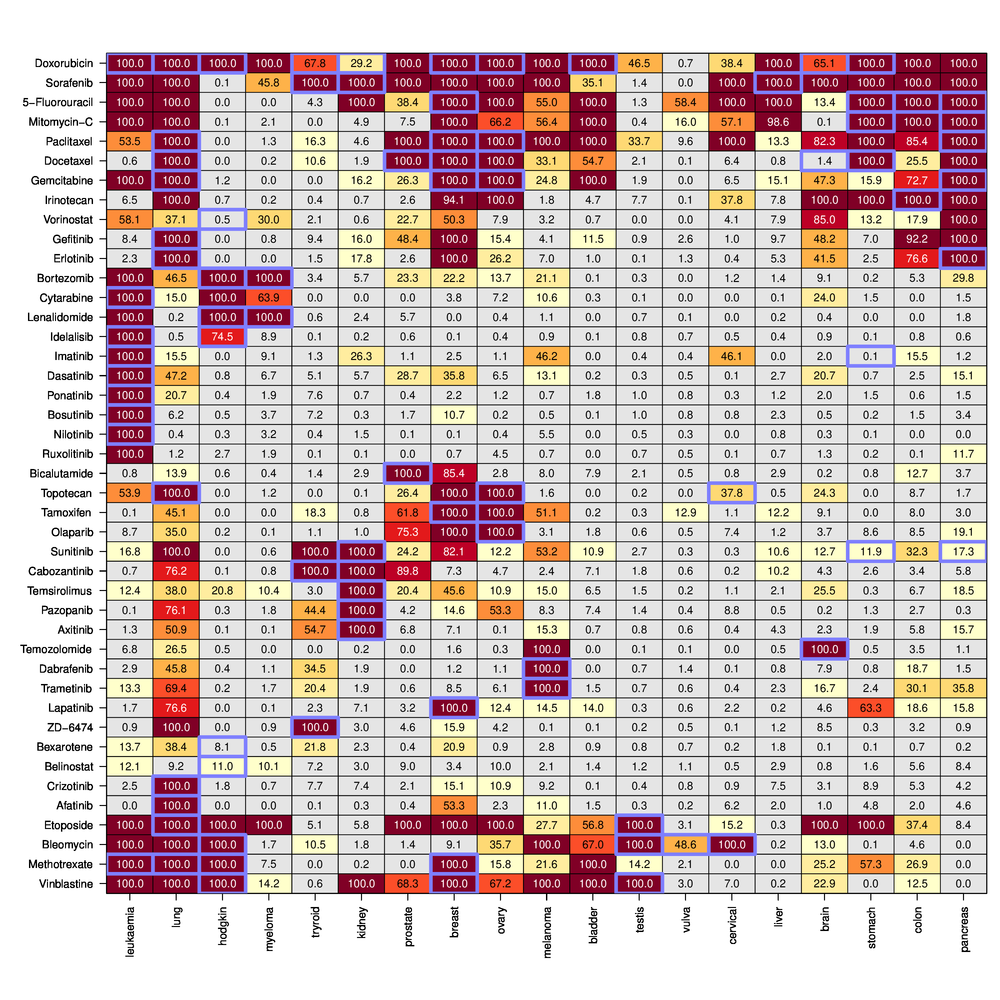

Supplement: Supplementary file 4 — Additional file 4: Figure S3. Heatmap visualizing the significance of the overlap between compounds (y-axis) and cancer types (x-axis). The overlap is assessed based on the number of common publications in comparison to the total number of publications ($-\text{log}_{10}$-log10 p-value of Fisher’s test truncated at 100). We selected only the compounds and cancer types with information about approval by the FDA. If the combination of compound is approved for a cancer type than a blue box is drawn. [file 12967_2021_2941_MOESM4_ESM.png]
